# Metastatic clear-cell renal cell carcinoma: a frequent *NOTCH1* mutation predictive of response to anti-NOTCH1 CB-103 treatment

**DOI:** 10.1186/s40164-023-00408-z

**Published:** 2023-05-15

**Authors:** Thi Oanh Bui, Eurydice Angeli, Morad El Bouchtaoui, Guillaume Gapihan, Van Tu Dao, Justine Paris, Christophe Leboeuf, Michael Soussan, Patrick Villarese, Marianne Ziol, Emmanuel Van Glabeke, Thi Huong Le, Jean-Paul Feugeas, Anne Janin, Guilhem Bousquet

**Affiliations:** 1 National Cancer Hospital, Cancer Research and Clinical Trials Center, Hanoi, Vietnam; 2 Université Paris Cité, INSERM, UMR_S942 MASCOT, F-75006 Paris, France; 3 Hanoi Medical University, Hanoi, Vietnam; 4 Université Sorbonne Paris Nord, 93439 Villetaneuse, France; 5 Assistance Publique Hôpitaux de Paris, Hôpital Avicenne, Service de Médecine Nucléaire, 93000 Bobigny, France; 6 Laboratoire d’Onco-Hématologie, Assistance Publique Hôpitaux de Paris, Hôpital Necker, 75015 Paris, France; 7 Assistance Publique Hôpitaux de Paris, Hôpital Avicenne, Service d’Anatomie Pathologique, 93000 Bobigny, France; 8 Fédération d’Urologie de Seine-Saint-Denis, 93100 Montreuil, France; 9 Université de Franche-Comté, 25000 Besançon, France; 10 Université de Paris, INSERM, U1137, F-75006 Paris, France; 11 Assistance Publique Hôpitaux de Paris, Hôpital Avicenne, Service d’Oncologie Médicale, 93000 Bobigny, France

**Keywords:** NOTCH1 mutation, Metastatic clear-cell renal cell carcinoma, Anti-NOTCH1 treatment, CB-103, Biomarker

## Abstract

**Background:**

Clear-cell renal cell carcinomas (ccRCCs) are malignant tumors with high metastatic potential and resistance to treatments occurs almost constantly. Compared to primary tumors, there are still limited genomic data that has been obtained from metastatic samples.

**Methods:**

We aimed to characterize metastatic ccRCC by way of whole-genome analyses of metastatic formalin-fixed samples, using OncoScan^®^ technology. We identified a frequent, unexpected pL1575P *NOTCH1* mutation which we set out to characterize for translational purposes. We thus implemented patient-derived xenografts from metastatic samples of human ccRCC to explore its clinical significance.

**Results:**

We showed that pL1575P *NOTCH1* mutation was an activating mutation, leading to the expression of NOTCH1-intracellular domain-active fragments in both cancer cells and tumor endothelial cells, suggesting a trans-differentiation of cancer cells into tumor micro-vessels. We demonstrated that this mutation could be used as a predictive biomarker of response to CB-103, a specific NOTCH1-intracellular domain inhibitor. One striking result was the considerable anti-angiogenic effect, coherent with the presence of *NOTCH1* mutation in tumor micro-vessels.

**Conclusions:**

We identified a frequent, unexpected pL1575P_c4724T_C *NOTCH1* mutation as a new biomarker for ccRCC metastases, predictive of response to the CB103 NOTCH1-intracellular domain inhibitor.

**Supplementary Information:**

The online version contains supplementary material available at 10.1186/s40164-023-00408-z.

**To the Editor,**

The incidence and deaths of kidney cancer worldwide, and thus mainly of clear-cell renal cell carcinoma (ccRCC), have doubled since three decades, particularly in the elderly population of countries with high social-demographic index [1]. Recent therapeutic advances have considerably improved the prognosis of metastatic ccRCCs. However, almost all patients develop resistance to these treatments [2]. Metastases may derive from minority clones in the heterogeneous primary tumor [3], and there are still limited genomic data obtained from metastases [4, 5].

Using OncoScan^®^ technology [6], we aimed to characterize metastatic ccRCCs by way of whole-genome analyses of metastatic formalin-fixed samples.

Four patients were included in the first analysis (Additional file 1: Table S1). We identified copy number alterations and loss of heterozygosity (LOH) (Additional file 1: Fig. S1A). We compared this data obtained from the recent meta-analysis we performed on ccRCCs (Additional file 1: Fig. S1B) [4] and found some abnormalities not previously described like 9q11.2 amplification (Additional file 1: Table S2). We also identified potential mutations (File ExcelS1). Using a threshold of 9 for probability score, we retrieved 48 mutations (Additional file 1: Table S3), several of them not yet described in ccRCC. We focused on the pL1575P_c4724T_C *NOTCH1* mutation, located on chromosome 9q34.3, because it was present in 3 of the 4 metastases, and because the NOTCH pathway is a potential therapeutic target in ccRCC [7]. In addition, the 9q arm is lost in 75% of RCCs (Additional file 1: Fig. S1B) [4], and we found an allelic imbalance 9q34.3 cytoband for Patients 1 and 2 (Additional file 1: File ExcelS1).

Using digital-droplet PCR (ddPCR) and specific probes for the pL1575P_c4724T_C *NOTCH1* mutation, we confirmed that it was present in the lymphoblastic acute leukemia T (LAL-T) positive control sample and in the 3 metastatic samples from Patients 1, 2 and 4 with high allele frequencies (Fig. 1A), but not in Patient 3. When we tested 9 additional ccRCC metastatic samples, we identified pL1575P *NOTCH1* mutations in all samples, with a mean mutant allele frequency of 53.5% (Fig. 1B).

**Fig. 1:**
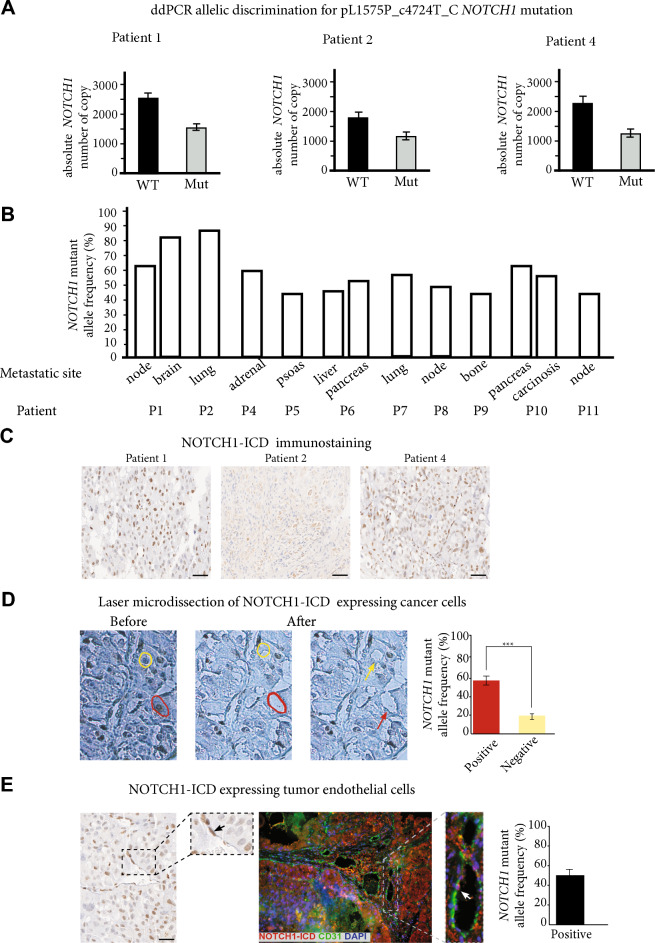
ddPCR allelic discrimination for the pL1575P_c4724T_C *NOTCH1* mutation, in 3 of the 4 metastatic samples processed via Oncoscan^®^ analysis (**A**), and in ten additional metastatic samples (**B**). NOTCH1-ICD-expressing cancer cells and tumor endothelial cells in human samples, NOTCH1-ICD staining is mainly nuclear in the 3 metastatic samples from Patients 1, 2 and 4 (**C**). **D** Panel B illustrates the laser-microdissection of a cancer cell expressing NOTCH1-ICD (red circle and arrow) and of a cancer cell not expressing NOTCH1-ICD (yellow circle and arrow), with a significant difference in terms of percentage of *NOTCH1* mutant allele frequency. *** *P* < 0.001. **E** The left panel shows the NOTCH1-ICD staining of a tumor endothelial cell (black arrow). Some tumor endothelial cells can be seen to co-express NOTCH1-ICD (red) and anti-CD31 (green) on immunofluorescence staining (middle panel). The right panel shows that laser-microdissected tumor endothelial cells expressing NOTCH1-ICD harbor the pL1575P *NOTCH1* mutation with an allelic frequency of 48%

Most *NOTCH1* mutations occur in the HD and/or PEST domains (Additional file 1: Fig. S2A). The pL1575P *NOTCH1* mutation is located in the HD-N domain and is responsible for NOTCH1 constitutive activation in LAL-T [8], through the release of the active NOTCH1 intracellular domain (NOTCH1-ICD) into the cytoplasm and its nucleus translocation (Additional file 1: Fig. S2B).

To identify NOTCH1-ICD, we performed immunostaining using an anti-NOTCH1 antibody (aa1755-1767-intracellular) which specifically recognizes an ICD epitope. We found predominant nuclear staining in the 3 metastatic samples from Patients 1, 2 and 4 (Fig. 1C), but not all cancer cells were stained. Using laser-microdissection, the pL1575P *NOTCH1* mutation was mainly present in cancer cells expressing NOTCH1-ICD (57% vs.17%, *P* < 0.01, Fig. 1D).

Surprisingly, we found that some CD31-expressing endothelial cells, but not all, co-expressed NOTCH1-ICD (Fig. 1E). Using laser-micro-dissection, we also identified the *NOTCH1* mutation with an allelic frequency of 48% (Fig. 1E), suggesting vascular mimicry [9].

Five patient-derived xenograft models obtained from ccRCC metastases were developed in our research unit (XRCC1 to XRCC5). We identified the *NOTCH1* mutation in all five models at Passage 1 (P1). Except for the XRCC5 model, the allelic frequency significantly increased between P1 and P5 (Additional file 1: Fig. S3). We chose XRCC4 and XRCC5 because of the marked enrichment for *NOTCH1* mutation in the XRCC4 xenograft (48% allelic mutation frequency at P5) and a much lower allele mutation frequency for the XRCC5 xenograft (19% at P5). XRCC4 and XRCC5 were obtained from patients responding to sunitinib (Additional file 1: Fig. S4), predicting response to sunitinib in the two models [10]. We treated them with two NOTCH1 inhibitors: LY411575, a γ-secretase inhibitor with an IC50 of 0.39 nM, and CB-103, a NOTCH1-ICD specific inhibitor (Additional file 1: Table S4 and Additional file 1: Fig. S2B). Using LY411575 administered daily by gavage, we did not observe any anti-tumor effect. In contrast, CB-103 mono-therapy induced a significant anti-tumor effect for both models, more marked with XRCC4 (Fig. 2A, Additional file 1: Table S5, Fig. S5A). Unexpectedly, like with sunitinib, there was also a strong induction of necrosis with CB-103 mono-therapy. The additive effect of the combined treatment was low in terms of tumor growth inhibition (Fig. 2B and Additional file 1: Fig. S5B), but tissue effects were much more marked than with sunitinib or CB-103 mono-therapy, in particular for necrotic area extent with very low cell viability. There was also a significant gradual decrease in micro-vessel density and proliferation (P < 0.01, Fig. 2C–D). CB-103 tumor growth inhibition was stronger with XRCC4 than with XRCC5 (Additional file 1: Table S5), coherent with a higher allelic mutation frequency in XRCC4. Finally, when we assessed NOTCH1-ICD expression and *NOCTH1* allelic mutation frequency in tumors after treatment, we found a significant decrease for both markers. Model XRCC4 treated with CB-103 monotherapy, *NOTCH1* allelic frequency decreased from 64 to 1%, and NOTCH1-ICD was no longer seen to be expressed on western blot (Fig. 2E, F).

**Fig. 2:**
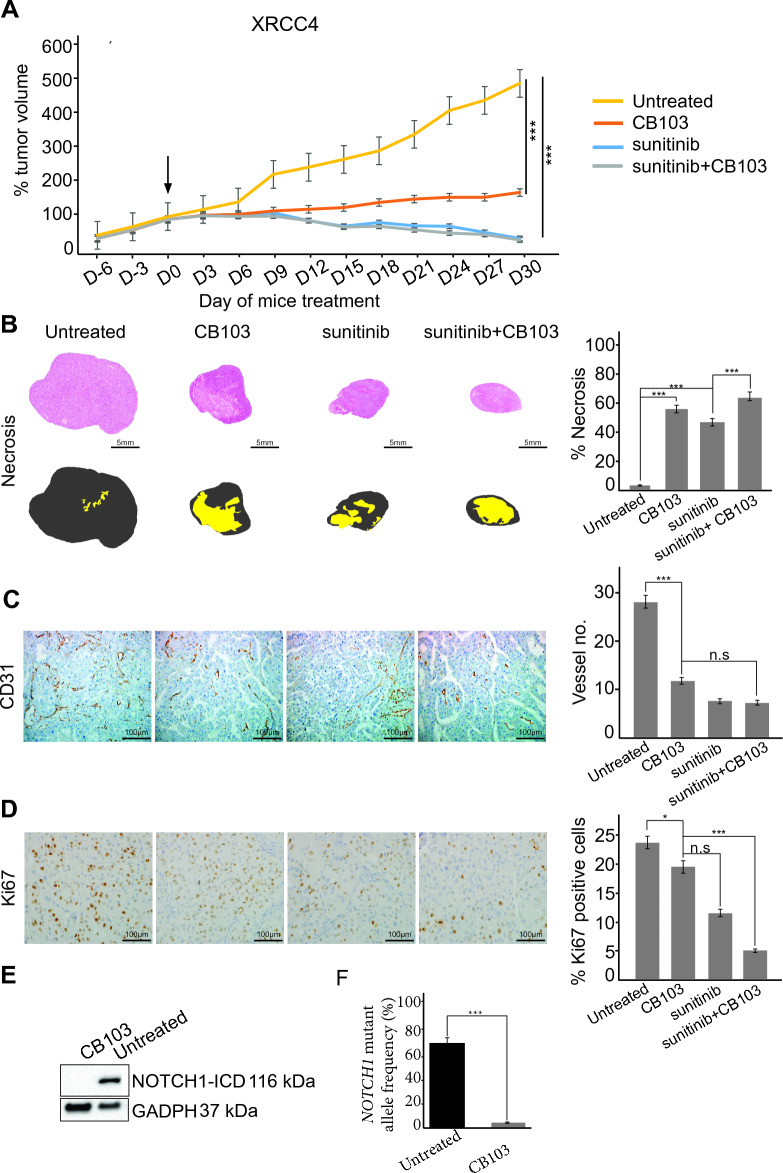
In vivo anti-tumor effect of CB-103 in a XRCC4 xenograft model. **A** CB-103 monotherapy, sunitinib monotherapy, or the combination of CB-103 and sunitinib significantly inhibit tunor growth after 30 days of treatment (n = 6 per treatment group). This is associated with a significant gradual increase in the percentage of necrotic areas (**B**), a significant decrease in microvessel density (**C**) and in cell proliferation (**D**). After 30 days of CB-103 monotherapy treatment, the pL1575P_c4724T_C *NOTCH1* mutation and NOTCH1-ICD protein expression disappear on Western blot (**E**). (*P < 0.05, ***P < 0.001) (**F**) NOTCH1 mutant allele frequency on laser-micro dissected cancer cells after treatment with CB103 or not, using ddPCR

Using two different methods, we showed that the pL1575P_c4724T_C *NOTCH1* activating mutation is frequent in metastatic ccRCCs, and this may be explained by a high sensitivity of Oncoscan^®^ technology using formalin-fixed samples [6, 11]. Like in LAL-T, we also evidenced the benefit of using CB-103 designed to block active forms of NOTCH1-ICD [12]. CB-103 is currently being evaluated in phase II clinical trials for various malignancies, with acceptable toxicity profile [13]. Since NOTCH signalling is closely linked to immune system regulation, further studies are required to determine if this *NOTCH1* mutation is associated with response to immune checkpoint inhibitors, which would be of high translational relevance [14]. Our study opens the way to further development in metastatic ccRCC.

## Supplementary Information

**Additional file 1: Fig S1**. Copy number gains and losses in 4 metastatic RCC samples using Oncoscan® (A) compared with 433 metastatic RCC samples from our meta-analysis on genomic data of clear-cell RCC (B). Some of the abnormalities were not previously described, including 9q11.2 and 15q11.1–11.2 amplifications (red arrows on panel A). **Fig S2.** NOTCH1 protein structure and signaling pathway. (A) NOTCH1 protein structure, the mature NOTCH1 receptor is a heterodimer composed of an extracellular subunit (NOTCH1-EC) and a transmembrane and intracellular subunit (NOTCH1-TMIC). NOTCH1-EC includes epidermal growth factor (EGF)-like repeats, involved in ligand binding, three LIN-12/NOTCH repeats (LNR), which prevent receptor activation in the absence of ligands, and the heterodimerization domain (HD) involved in non-covalent interactions between NOTCH1-EC and NOTCH1-TMIC. NOTCH1-TMIC comprises the transmembrane domain (TM) and the intracellular domain (ICD) (NOTCH1-ICD). NOTCH1-ICD comprises an RBPJ-associated molecule (RAM) domain, seven ankyrin (ANK) repeats, nuclear localization signals (NLS), a transactivation domain (TAD), and a PEST domain, in turn involved in proteasomal degradation of active NOTCH1-ICD. Most NOTCH1 mutations are located in the HD and PEST domains (red arrows), and the pL1575P_c4724T_C NOTCH1 mutation (highlighted in yellow) is located in the HDN domain. (B) Newly synthesized NOTCH1 precursor is cleaved by a furin-like convertase (Furin) in the Golgi apparatus to generate the mature receptor. NOTCH1 signaling occurs when a JAGGED or DELTA ligand expressed on a signal-sending cell interacts with NOTCH1 on a signalreceiving cell. This interaction triggers two sequential cleavages of NOTCH1: the first, by way of an a disintegrin and metalloproteinase (ADAM) metalloproteinase, generates the substrate for the second cleavage by γ-secretase, which releases the active NOTCH1-ICD. NOTCH1-ICD translocates to the nucleus where it forms a transcriptional activation complex by interacting with the transcription factor CSL/RBP-Jk, mastermind-like proteins, and other coactivators (CoA), leading to the expression of NOTCH1 target genes. In physiological conditions, NOTCH1 expression is controlled by ubiquitination and proteasomal degradation of NOTCH1-ICD. **Fig S3**. ddPCR allelic discrimination for the pL1575P_c4724T_C NOTCH1 mutation in 5 XRCC tumor xenografts at the first (in gray) and the fifth (in black) passages. **Fig S4**. Response to sunitinib treatment in first-line setting for two patients who provided metastatic samples for xenograft in murine models. (A) Patient corresponding to the XRCC4 model. (B) Patient corresponding to the XRCC5 model. **Fig S5**. In vivo anti-tumor effect of CB-103 in the XRCC5 xenograft model. (A) CB-103 monotherapy, sunitinib monotherapy, or the combination of CB-103 and sunitinib significantly inhibit tumor growth after 30 days of treatment (n = 6 per treatment group). This is associated with a significant gradual increase in the percentage of necrotic areas (B), and significant decrease in microvessel density (C). For cell proliferation, the decrease is only significant with the combination of CB-103 and sunitinib compared to untreated mice (C). After 30 days of treatment with CB-103 monotherapy, the pL1575P_c4724T_C NOTCH1 mutation (D) has virtually disappeared. (*P < 0.05, ***P < 0.001). **Fig S6**. Oncoscan® technology and the Molecular Inversion Probe: Target Generation and Hybridization Procedures. a) Annealing: Probe and gDNA hybridization; b) Gap filling with A/T or G/C nucleotides; c) Exonuclease selection for gap filled probes; d) Cleavage at site 1 for probe opening and inversion; e) Probe amplification and biotinylation; f) Cleavage at site 2 to release the tag sequence; f) Array hybridization followed by staining with phycoerythrin through the biotin-streptavidin interaction; g) Array scanning. Blue and gray colors indicate presence and absence of the phycoerythrin fluorescence signal respectively. **Fig S7**. Tumor growth inhibition coefficient in XRCC models. For a drug or a drug combination, the coefficient of inhibition is calculated as (a’-a)/a, a being the slope of the curve before the start of treatment (Day 0), and a’ the slope of the curve between Day 0 and Day 30 of treatment. If this growth inhibition coefficient is found to be less than 0, the tumor is considered sensitive to the drug administered; if it is above 0, the tumor is considered resistant to the drug. **Table S1.** Characteristics of the four patients included in the initial study using Oncoscan® technology. **Table S2.** List of genes located in locus 9p11.2, 15q11.1, and 15q11.2. **Table S3.** Point mutation identified on RCC metastases before treatment. **Table S4.** NOTCH1 targeted therapeutics. **Table S5.** Growth inhibition coefficient for drugs tested in XRCC model. **Table S6.** Characteristics of the five patient-derived xenograft models.

## Data Availability

The gene expression data generated during the study are part of the supplementary information. All other datasets generated during the study will be made available upon reasonable request to the corresponding author, Prof. Guilhem Bousquet, email address: guilhem.bousquet@aphp.fr.

## Abbreviations

ccRCC: Clear-cell rencal cell carcinoma
LAL-T: Lymphoblastic acute leukemia T
XRCC: Xenograft renal cell carcinoma
FFPE: Formalin-Fixed Paraffin-Embedded

## References

[1] Bai X, Yi M, Dong B, Zheng X, Wu K (2020). The global, regional, and national burden of kidney cancer and attributable risk factor analysis from 1990 to 2017. Exp Hematol Oncol.

[2] Rini BI, Plimack ER, Stus V, Gafanov R, Hawkins R, Nosov D (2019). Pembrolizumab plus axitinib versus sunitinib for advanced renal-cell carcinoma. N Engl J Med.

[3] Gerlinger M, Horswell S, Larkin J, Rowan AJ, Salm MP, Varela I (2014). Genomic architecture and evolution of clear cell renal cell carcinomas defined by multiregion sequencing. Nat Genet.

[4] Bui TO, Dao VT, Nguyen VT, Feugeas J-P, Pamoukdjian F, Bousquet G (2022). Genomics of clear-cell renal cell carcinoma: a systematic review and meta-analysis. Eur Urol.

[5] Turajlic S, Xu H, Litchfield K, Rowan A, Chambers T, Lopez JI (2018). Tracking cancer evolution reveals constrained routes to metastases: TRACERx renal. Cell.

[6] Wood HM, Foster JM, Taylor M, Tinkler-Hundal E, Togneri FS, Wojtowicz P (2017). Comparing mutation calls in fixed tumour samples between the affymetrix OncoScan® array and PCR based next-generation sequencing. BMC Med Genomics.

[7] Bhagat TD, Zou Y, Huang S, Park J, Palmer MB, Hu C (2017). Notch pathway is activated via genetic and epigenetic alterations and is a therapeutic target in clear cell renal cancer. J Biol Chem.

[8] Malecki MJ, Sanchez-Irizarry C, Mitchell JL, Histen G, Xu ML, Aster JC (2006). Leukemia-associated mutations within the NOTCH1 heterodimerization domain fall into at least two distinct mechanistic classes. Mol Cell Biol.

[9] Serova M, Tijeras-Raballand A, Dos Santos C, Martinet M, Neuzillet C, Lopez A (2016). Everolimus affects vasculogenic mimicry in renal carcinoma resistant to sunitinib. Oncotarget.

[10] Varna M, Gapihan G, Feugeas J-P, Ratajczak P, Tan S, Ferreira I (2015). Stem cells increase in numbers in perinecrotic areas in human renal cancer. Clin Cancer Res.

[11] Alexiev BA, Zou YS (2014). Clear cell papillary renal cell carcinoma: a chromosomal microarray analysis of two cases using a novel Molecular Inversion Probe (MIP) technology. Pathol Res Pract.

[12] Lehal R, Zaric J, Vigolo M, Urech C, Frismantas V, Zangger N (2020). Pharmacological disruption of the Notch transcription factor complex. Proc Natl Acad Sci USA.

[13] Jose e P-G, Javier C, Anastasios s S, Rogier M, Elena L-M, Analía A, et al. First-in-human phase 1–2A study of CB-103, an oral Protein-Protein Interaction Inhibitor targeting pan-NOTCH signalling in advanced solid tumors and blood malignancies. ASCO annual meeting 2018. 2018. https://www.cellestia.com/wp-content/uploads/2021/05/7.1-ASCO-Poster_2018_Cellestia.pdf

[14] Zhou B, Lin W, Long Y, Yang Y, Zhang H, Wu K (2022). Notch signaling pathway: architecture, disease, and therapeutics. Sig Transduct Target Ther.

