# Supplementary material for: Metastatic clear-cell renal cell carcinoma: a frequent NOTCH1 mutation predictive of response to anti-NOTCH1 CB-103 treatment
Source: Exp Hematol Oncol. 2023 May 15;12:46. doi: 10.1186/s40164-023-00408-z (PMC10184347; doi:10.1186/s40164-023-00408-z)
Supplement: Supplementary file 1 — Additional file 1: Fig S1. Copy number gains and losses in 4 metastatic RCC samples using Oncoscan® (A) compared with 433 metastatic RCC samples from our meta-analysis on genomic data of clear-cell RCC (B). Some of the abnormalities were not previously described, including 9q11.2 and 15q11.1–11.2 amplifications (red arrows on panel A). Fig S2. NOTCH1 protein structure and signaling pathway. (A) NOTCH1 protein structure, the mature NOTCH1 receptor is a heterodimer composed of an extracellular subunit (NOTCH1-EC) and a transmembrane and intracellular subunit (NOTCH1-TMIC). NOTCH1-EC includes epidermal growth factor (EGF)-like repeats, involved in ligand binding, three LIN-12/NOTCH repeats (LNR), which prevent receptor activation in the absence of ligands, and the heterodimerization domain (HD) involved in non-covalent interactions between NOTCH1-EC and NOTCH1-TMIC. NOTCH1-TMIC comprises the transmembrane domain (TM) and the intracellular domain (ICD) (NOTCH1-ICD). NOTCH1-ICD comprises an RBPJ-associated molecule (RAM) domain, seven ankyrin (ANK) repeats, nuclear localization signals (NLS), a transactivation domain (TAD), and a PEST domain, in turn involved in proteasomal degradation of active NOTCH1-ICD. Most NOTCH1 mutations are located in the HD and PEST domains (red arrows), and the pL1575P_c4724T_C NOTCH1 mutation (highlighted in yellow) is located in the HDN domain. (B) Newly synthesized NOTCH1 precursor is cleaved by a furin-like convertase (Furin) in the Golgi apparatus to generate the mature receptor. NOTCH1 signaling occurs when a JAGGED or DELTA ligand expressed on a signal-sending cell interacts with NOTCH1 on a signalreceiving cell. This interaction triggers two sequential cleavages of NOTCH1: the first, by way of an a disintegrin and metalloproteinase (ADAM) metalloproteinase, generates the substrate for the second cleavage by γ-secretase, which releases the active NOTCH1-ICD. NOTCH1-ICD translocates to the nucleus where it forms a transcriptional activat [file 40164_2023_408_MOESM1_ESM.pdf]

Supplementary Methods

Patient metastatic samples processed for OncoScan® analysis

Four patients were included in the first analysis. They all had a metastatic clear-cell renal cell carcinoma (ccRCC) with a biopsy of a metastatic site performed before any medical treatment. All four metastatic samples processed for genomic analyses were formalin-fixed paraffin-embedded biopsy samples.

In compliance with the French bioethics legislation (2004-800; June 8, 2004), all patients had been informed of the research use of part of their samples remaining after diagnosis, and none opposed it. Informed consent was obtained from each patient to perform whole genome analyses on their tumor sample. The Clinical Research Board Ethics Committee approved this study (CPP-Ile-de-France#13218).

DNA purification and whole-genome analysis on human samples

Whole genome analysis was performed on laser-micro-dissected tumor cells from the four metastatic samples using OncoScan-Express® (Affymetrix, USA). OncoScan-Express® uses a molecular inversion probe (MIP)-based genotyping system dedicated to formalin-fixed paraffin-embedded tissues, which determines the genotype of 330,000 SNPs, copy number alterations, loss of heterozygosity (LOH) and somatic mutations (Supplementary Fig. 6). MIP probes are circularizable oligonucleotides, the two ends of which carry two sequences complementary to two sequences on the genome, separated by one nucleotide (where the variant to be genotyped is located). After hybridization of the genomic DNA, the product of the reaction is divided between two tubes, and two nucleotides are added to each tube (A/T and C/G). In the tube including the nucleotide complementary to the allele on the genome, the MIP probe is ligated and becomes circular. This structure is selected using exonucleases, and linearized. The products are amplified and hybridized onto an Affymetrix microarray for

product identification. At the time we performed the analysis, OncoScan® Assay searched for 541 somatic mutations specific to cancer, with a coverage of over 200 tumor suppressors and oncogenes, and a median spacing of 1 probe per 0.5 kb for the top 10 "actionable" tumor suppressor genes, a median spacing of 1 probe per 2 kb for the top 190-plus actionable oncogenes, and a median backbone spacing of 1 probe per 9 kb.

Data analyses were performed on Nexus-7 software (BioDiscovery,USA).

ddPCR and validation of the *NOTCH1* mutation in metastatic samples

A Droplet Digital Polymerase Chain Reaction (ddPCR) was performed using the QX100 ddPCR workflow system (Biorad, Hercules, CA, USA). The mix contained 20 ng of genomic DNA, 10 µL of ddPCR Supermix for Probes (No dUTP) (Bio Rad), 1µL of *NOTCH1* probes either wild-type or specific to the pL1575P *NOTCH1* mutation (12772, Qiagen, Germany) (Supplementary Word 1) and 1 µL *RNaseP* probe (Taqman® copy number Reference Assay, 4403326, Life Technologies) per well, and the final volume for the reaction was 20 µL. Droplets were generated by a QX200 Droplet Generator (Biorad). PCR was carried out on the CFX96 Real Time System (Bio Rad). It consisted in an initial denaturing step at 95 °C for 10 min., followed by 40 denaturing cycles (94 °C for 30 s), and annealing (60°C for 1 min.). A post-amplification melting curve program was initiated by heating to 98 °C for 10 min. and then cooling to 12 °C. Each PCR run included a no-template control. The results for ddPCR were generated using QX100 Droplet Reader (Biorad) and analysed using QuantaSoft software (Biorad). The ratio of *NOTCH1*-positive droplets to *RnaseP*-positive droplets was calculated.

We calculated the mutant allele frequency using the mathematical formula Mmu/ MDNAconc, as previously reported, Mmu being the number of mutant copies per droplet and MDNAconc being the DNA concentration in the reaction. Mmu and MDNAconc are calculated as follows:

Mmu = -ln (1-(nmu/n)), where nmu = number of droplets positive for mutant *NOTCH1* probe and n = total number of droplets. MDNAconc = -ln (1-(nDNAcon/n)), where nDNAconc = number of droplets positive for wild-type *NOTCH1* probe and/or mutant *NOTCH1* probe and n = total number of droplets [1].

A DNA obtained from a patient with lymphoblastic acute leukaemia T and the presence of pL1575P *NOTCH1* mutation was provided by P.V and used as the positive control.

Detection of NOTCH1-ICD-expressing cancer cells in metastases and tumor xenografts

Immunohistochemistry staining

An indirect immunoperoxidase method was performed on 5µm-thick tissue sections using anti-NOTCH1-ICD (ab8925,1:200, Abcam) as primary antibody.

The systematic controls used were the absence of primary antibody and the use of an irrelevant primary antibody of the same isotype.

Immunofluorescence staining

To detect NOTCH1-ICD in the endothelial cells, a double indirect immunofluorescence method was performed using NOTCH1-ICD and CD31 as primary antibodies. Staining was performed with Tyramide detection kit 488CF (biotium, 99824), and 543CF (biotium, 99825) respectively, and a fluorescent mounting medium with DAPI was used for nucleus detection (E19-18, GBI labs).

Western-blot analysis

Protein extraction was performed from cryopreserved tissue with RIPA buffer (Thermo) supplemented with 1 EDTA-free protease inhibitor cocktail tablet (Roche Diagnostics) and phosphatase inhibitors (Sigma-Aldrich).

Western blot was performed on 10% Mini-PROTEAN TGX precast gels (Biorad), then transferred onto a 0.2 µm Nitrocellulose membrane (Biorad) using the Trans-Blot Turbo Transfer System. Immunostaining was performed using anti-NOTCH1-ICD (ab8925, 1:500,

Abcam) and anti-GAPDH (ab9485, 1:2500, Abcam) as primary antibodies. An anti-rabbit HRP (ab32568, 1:1000, Abcam) was used as a second antibody. Western blot revelation was performed using Clarity Western ECL Substrate (Biorad) and detected on ChemiDoc XRS+ detection system (Biorad). Analyses were performed with Image Lab Software 6.1 (Biorad).

Laser micro-dissection of NOTCH1-ICD expressing cells

To confirm that NOCTH1-ICD expression in cancer cells or tumor endothelial cells was associated with *NOTCH1* mutation, we combined laser micro-dissection of NOTCH1-ICD expressing cells with ddPCR for *NOTCH1* mutation.

For each sample analysed, 7 µm-thick tissue sections were laser-micro-dissected to select a minimum of 300 cancer cells or 100 tumor endothelial cells expressing NOCTH1-ICD, using a PALM-Microbeam/Zeiss-system (Carl Zeiss, Germany). Total DNA was extracted from the micro-dissected cells using DNeasy-Micro-Kit (Qiagen, Courtaboeuf, France), and concentrated in a final volume of 10 µL.

For *NOTCH1* gene copy number analyses, total DNA extracted from micro-dissected tumor cells was processed using ddPCR as described above.

Patient-derived clear-cell renal cell carcinoma xenografts and treatment

Five patient-derived clear-cell RCC xenograft models obtained from biopsies of metastases were implemented in our research unit [2] (Supplementary Table 6). The National Ethics Committee for experimental animal studies approved this study (APAFIS#17190-2018101814245111). The ethical guidelines that were followed met the standards required by the US guidelines [3]. Animals were maintained in pathogen-free housing at Sorbonne Paris Nord University (agreement number: C9300801).

After successful engraftment, tumor growth was measured in two perpendicular diameters with a caliper. Tumor volumes were calculated as follows: V = L × l2 ÷ 2, L being the larger diameter (length), l the smaller (width). When tumors reached a volume of 400 mm3 (n = 6

mice per treatment group), the mice were separated into four groups: i) treatment by gavage with sunitinib at 40 mg/kg/day (group 1), ii) treatment by gavage with NOTCH1 inhibitor (CB-103, HY-135145, MedChem Express at 25 mg/kg/twice daily or (LY411575, S2714, Selleckchem) at 10 mg/kg/day (group 2)); iii) combined treatment by gavage with sunitinib, at 40 mg/kg/day, and with NOTCH1 inhibitor (either CB-103 or LY411575) (group 3); iv) treatment with 100 µL of 0.9% NaCl as a control (group 4). All treatments were administered for 30 days. A daily clinical score was recorded and tumor growth was measured weekly until tumor weight reached the ethically recommended limit of under 10 % of mouse body weight (Directive 2010/63/EU of European Parliament and Council of 22 September 2010 on the protection of animals used for scientific purposes; Official Journal of European Union L 276/33). An inhibition growth coefficient was calculated for each treatment arm (Supplementary Fig. 7), using a ratio of the slopes (a and a') of the straight lines before and after treatment. In all xenograft models, the coefficient of inhibition for a drug was calculated as (a'-a)/a - a, being the slope of the curve before the start of treatment (Day 0) - and a' the slope of the curve between Day 0 and Day 30 of treatment. For a given drug combination, if the inhibition coefficient was negative, the tumor was considered sensitive to the drug. If, in contrast, it was positive, the tumor was considered resistant to this drug.

***In situ* assessment of necrosis, cell proliferation, angiogenesis and apoptosis**

When present, necrosis was delineated on virtual slides created on a Nanozoomer2.0H scanner (Hamamatsu/ Japan), and quantified using DotSlide2 software. Results were expressed as the sum of necrotic areas for each section, and the mean ± SEM. For micro-vessel density, proliferation and apoptotic counts, an indirect immunoperoxidase method was performed on 5 µm-thick tissue sections, using monoclonal mouse anti-human Ki67 antibody (M724001-2, 1:100, Agilent) as primary antibody for proliferation, polyclonal rabbit anti-human cleaved-caspase-3 antibody (Asp175, 1:200, Cell signalling Technology) as primary

antibody for apoptosis, and rabbit polyclonal anti-mouse CD31 antibody (SZ31, 1:30, Dianova) as primary antibody for micro-vessel density. For CD31 staining, the secondary antibody was a rabbit anti-rat IgG H&L (ab6703,1:200, Abcam), for Ki67 staining, a rabbit anti-mouse IgG1 H&L (clone M1gG51-4, 1:200, Abcam) was used. The secondary antibody was coupled with an anti-rabbit OmniMap detection kit (Roche diagnostic, Meylan, France).

For each tumor section analyzed, proliferation and apoptotic cell counts were performed on five different fields at x400 magnification, using a ProvisAX70 microscope (Olympus, Tokyo) with a wide-field eyepiece number 26.5 providing a field size of 0.344mm2 at X400 magnification. Microscope images were captured using a ColorView-III digital camera, and analyzed using Olympus-SIS Cell F software. The percentage of positive cells in 100 cancer cells was determined, and results were expressed as means ± SEM. For micro-vessel density, CD31-positive micro-vessels were counted on ten different fields, at X400 magnification. The number of positive micro-vessels was related to the total number of micro-vessels in a given surface area studied. Results were expressed as means ± standard deviation.

Statistical analysis

Statistical analyses were performed using R Studio 1.3.1073 statistical software.

For analysis of the copy number variation derived from the result from Oncoscan® technology and our meta-analysis data, we used the *copynumber* package in R.

For counts of NOTCH1-expressing tumor cells, the mean ±SEM was calculated for each tumor sample (primary RCC, metastasis or tumor-xenograft), and represented in bar graphs.

Quantitative values were compared using Student's t-test (two-tailed), while proportions were compared using the Z-test. P values under 0.05 were considered significant.

References

1. Hrebien S, O'Leary B, Beaney M, Schiavon G, Fribbens C, Bhambra A, et al. Reproducibility of Digital PCR Assays for Circulating Tumor DNA Analysis in Advanced Breast Cancer. Mazoyer S, editor. PLoS ONE. 2016;11:e0165023.

2. Bousquet G, El Bouchtaoui M, Sophie T, Leboeuf C, de Bazelaire C, Ratajczak P, et al. Targeting autophagic cancer stem-cells to reverse chemoresistance in human triple negative breast cancer. Oncotarget. 2017;8:35205–21.

3. U.S. Department of Health and Human Services National Institutes of Health Office of Laboratory Animal Welfare. Public Health Service Policy on Humane Care and Use of Laboratory Animals. NIH Publication revised; 2015.

4. Motzer RJ, Mazumdar M, Bacik J, Berg W, Amsterdam A, Ferrara J. Survival and Prognostic Stratification of 670 Patients With Advanced Renal Cell Carcinoma. JCO. 1999;17:2530–2530.

Figure legends

Supplementary Fig. 1. Copy number gains and losses in 4 metastatic RCC samples using Oncoscan® (A) compared with 433 metastatic RCC samples from our meta-analysis on genomic data of clear-cell RCC (B). Some of the abnormalities were not previously described, including 9q11.2 and 15q11.1-11.2 amplifications (red arrows on panel A).

Supplementary Fig. 2. NOTCH1 protein structure and signaling pathway. (A) NOTCH1 protein structure, the mature NOTCH1 receptor is a heterodimer composed of an extracellular subunit (NOTCH1-EC) and a transmembrane and intracellular subunit (NOTCH1-TMIC). NOTCH1-EC includes epidermal growth factor (EGF)-like repeats, involved in ligand binding, three LIN-12/NOTCH repeats (LNR), which prevent receptor activation in the absence of ligands, and the heterodimerization domain (HD) involved in non-covalent interactions between NOTCH1-EC and NOTCH1-TMIC. NOTCH1-TMIC comprises the transmembrane domain (TM) and the intracellular domain (ICD) (NOTCH1-ICD). NOTCH1-ICD comprises an RBPJ-associated molecule (RAM) domain, seven ankyrin (ANK) repeats, nuclear localization signals (NLS), a transactivation domain (TAD), and a PEST domain, in turn involved in proteasomal degradation of active NOTCH1-ICD. Most NOTCH1 mutations are located in the HD and PEST domains (red arrows), and the pL1575P_c4724T_C NOTCH1 mutation (highlighted in yellow) is located in the HDN domain. (B) Newly synthesized NOTCH1 precursor is cleaved by a furin-like convertase (Furin) in the Golgi apparatus to generate the mature receptor. NOTCH1 signaling occurs when a JAGGED or DELTA ligand expressed on a signal-sending cell interacts with NOTCH1 on a signal-receiving cell. This interaction triggers two sequential cleavages of NOTCH1: the first, by way of an a disintegrin and metalloproteinase (ADAM) metalloproteinase, generates the substrate for the second cleavage by γ-secretase, which releases the active NOTCH1-ICD. NOTCH1-ICD translocates to the nucleus where it forms a transcriptional activation complex

by interacting with the transcription factor CSL/RBP-Jk, mastermind-like proteins, and other coactivators (CoA), leading to the expression of NOTCH1 target genes. In physiological conditions, NOTCH1 expression is controlled by ubiquitination and proteasomal degradation of NOTCH1-ICD.

Supplementary Fig. 3. ddPCR allelic discrimination for the pL1575P_c4724T_C NOTCH1 mutation in 5 XRCC tumor xenografts at the first (in gray) and the fifth (in black) passages.

Supplementary Fig. 4. Response to sunitinib treatment in first-line setting for two patients who provided metastatic samples for xenograft in murine models. (A) Patient corresponding to the XRCC4 model. (B) Patient corresponding to the XRCC5 model.

Supplementary Fig. 5. In vivo anti-tumor effect of CB-103 in the XRCC5 xenograft model. (A) CB-103 monotherapy, sunitinib monotherapy, or the combination of CB-103 and sunitinib significantly inhibit tumor growth after 30 days of treatment (n = 6 per treatment group). This is associated with a significant gradual increase in the percentage of necrotic areas (B), and significant decrease in microvessel density (C). For cell proliferation, the decrease is only significant with the combination of CB-103 and sunitinib compared to untreated mice (C). After 30 days of treatment with CB-103 monotherapy, the pL1575P_c4724T_C NOTCH1 mutation (D) has virtually disappeared. (*P < 0.05, ***P < 0.001).

Supplementary Fig. 6. Oncoscan® technology and the Molecular Inversion Probe: Target Generation and Hybridization Procedures. a) Annealing: Probe and gDNA hybridization; b) Gap filling with A/T or G/C nucleotides; c) Exonuclease selection for gap filled probes; d) Cleavage at site 1 for probe opening and inversion; e) Probe amplification and biotinylation; f) Cleavage at site 2 to release the tag sequence; f) Array hybridization followed by staining with phycoerythrin through the biotin-streptavidin interaction; g) Array scanning. Blue and

gray colors indicate presence and absence of the phycoerythrin fluorescence signal respectively.

Supplementary Fig. 7. Tumor growth inhibition coefficient in XRCC models. For a drug or a drug combination, the coefficient of inhibition is calculated as (a’-a)/a, a being the slope of the curve before the start of treatment (Day 0), and a’ the slope of the curve between Day 0 and Day 30 of treatment. If this growth inhibition coefficient is found to be less than 0, the tumor is considered sensitive to the drug administered; if it is above 0, the tumor is considered resistant to the drug.

Supplementary Table 1: Characteristics of the four patients included in the initial study using Oncoscan® technology

	Age at the time of surgical sampling	Histological type	Furhman grade	Risk group*	Metastatic localizations	Site of surgical sampling
Patient 1	57	Clear-cell	3	Intermediate	Brain, lung, lymph nodes	Lung
Patient 2	80	Clear-cell	3	Good	Lung	Lung
Patient 3	60	Clear-cell	3	Intermediate	Lung	Lung
Patient 4	54	Clear-cell	3	Good	Adrenal, brain	Adrenal

* According to MSKCC classification [4]

Supplementary Figure 1

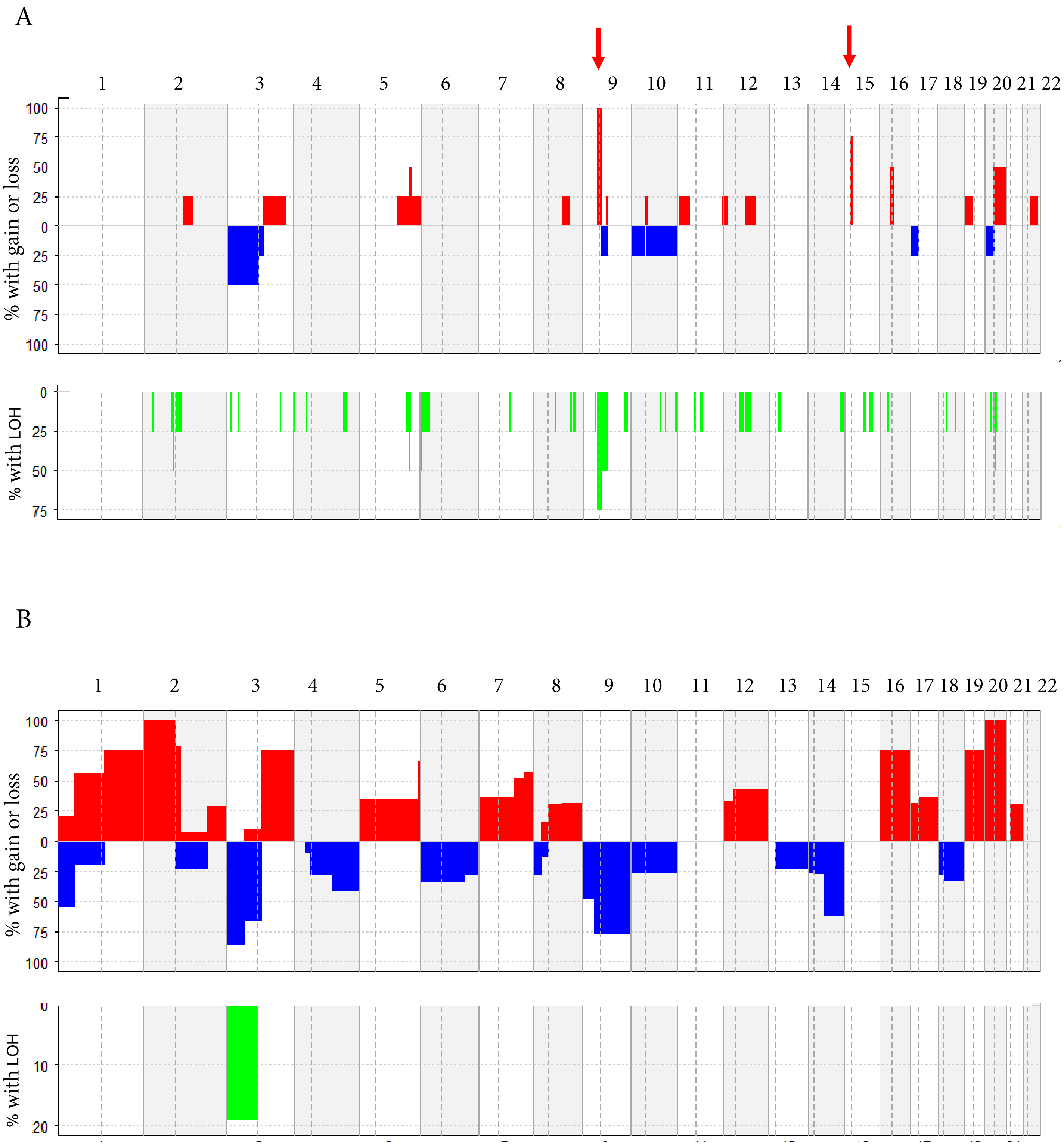

Supplementary Table 2. List of genes located in locus 9p11.2, 15q11.1, and 15q11.2

ID	Locus	Gene	Reference	Function in renal cancer
1	9p11.2	*MIR1299*	1. Lin., *J Cell Biochem.*, 2020	MIR1299 encodes the acting circRNA and circ-EGLN3, which promotes renal cell carcinoma proliferation by regulating of IRF7 expression
2		*CNTNAP3B*		Unknown
3		*FOXD4L6*		Unknown
4		*CBWD6*		Unknown
5		*PGM5P2*		Unknown
6		*SPATA31A6*		Unknown
7		*ANKRD20A2P*		Unknown
8		*MEP1AP1*		Unknown
9		*PTS-P1*		Unknown
10		*FAM27E2*		Unknown
11		*FAM95B1*		Unknown
12		*GXYLT1P3*		Unknown
13		*MIR4477A*		Unknown
14		*BMS1P14*		Unknown
15		*FRG1HP*		Unknown
16		*RPL7AP45*		Unknown
17		*LOC100132004*		Unknown
18		*LINC00268*		Unknown
19		*RNA5SP530*		Unknown
20		*RN7SL565P*		Unknown
21		*RN7SL343P*		Unknown
22		*IGKV1OR9-2*		Unknown
23		*RNU6-1269P*		Unknown
24		*RNU6-156P*		Unknown
25		*FAM242F*		Unknown
26		*AQP7P5*		Unknown
27		*LOC102724580*		Unknown
28		*CDRT15P14*		Unknown
29		*CDRT15P6*		Unknown
30		*SNX18P8*		Unknown
31		*CDK2AP2P1*		Unknown
32		*RBM17P3*		Unknown
33		*VN2R6P*		Unknown
34		*FAM27D1*		Unknown
35		*CNN2P2*		Unknown
36		*MYO5BP1*		Unknown
37		*PTGER4P1*		Unknown
38		*LOC440896*		Unknown
39		*LOC107984035*		Unknown
40		*LOC112268035*		Unknown
41		*LOC112268034*		Unknown
42		*LOC112268033*		Unknown

43		*LOC107987000*		Unknown
44		*LOC107986999*		Unknown
45		*LOC107986998*		Unknown
46		*LOC107986997*		Unknown
47		*LOC107986995*		Unknown
48		*LOC107986993*		Unknown
49		*LOC107986992*		Unknown
50		*LOC107986991*		Unknown
51		*LOC107984006*		Unknown
52		*LOC105376065*		Unknown
53		*LOC105376063*		Unknown
54		*LOC105376060*		Unknown
55		*LOC105376058*		Unknown
56		*LOC105376057*		Unknown
57		*LOC105369249*		Unknown
58		*LOC103047893*		Unknown
59		*LOC102724431*		Unknown
60		*LOC101928906*		Unknown
61		*LOC100421691*		Unknown
62		*LOC100420440*		Unknown
63		*LOC100419924*		Unknown
64		*LOC100419690*		Unknown
65	15q11.1			Unknown
66		*CHEK2P2*		Unknown
67		*IGHV1OR15-9*		Unknown
68		*BMS1P15*		Unknown
69		*IGHV1OR15-2*		Unknown
70		*IGHV3OR15-7*		Unknown
71		*RHPN2P1*		Unknown
72		*SLC20A1P3*		Unknown
73		*RN7SL584P*		Unknown
74		*RNU6-978P*		Unknown
75		*FAM30B*		Unknown
76		*BCAR1P1*		Unknown
77		*LOC105379203*		Unknown
78		*LOC646071*		Unknown
79	15q11.2	*UBE3A*	1. Wu, Genet Mol Res., 2016	UBF3A (known as E6AP) is an enzyme that is involved in protein targeting for degradation
80		*SNRPN*	1. Jiang., Front Genet., 2021	SNRPN is one of the RNA-binding proteins, with a role in the post-transcriptional regulation of gene expression. These RNA-binding proteins are related to known cancer biomarkers (such as BCL2, promoting ovarian cancer cell survival…). Thus, dysfunctional RNA-binding protein can lead to oncogenenis in pRCC.
81			2. Munroe, Front Immunol., 2021	
82			3. Schweinfest., Cancer Res., 1997	
83			4. Schuster., Lab Invest., 2003	
84			5. Larsson., Cell., 1995	

85			6. Richards., J Exp Med., 1998	
86			7. Kondo., Oncogene., 1995	
87		*SNHG14*	1. Liu., Am J Cancer Res., 2017	SNHG14 is a long non-coding RNA, that promotes ccRCC cell migration and invasion by acting as a competing endogenous RNA.
88			2. Wang., BMC Nephrol., 2020	
89		*PWRN1*	1. Qing., Mol Med Rep., 2019	PWRN1 is a long non-coding RNA, that promotes pRCC cell migration and invasion by acting as a competing endogenous RNA.
90		*DEL15Q11.2*		Unknown
91		*MKRN3*		Unknown
92		*CYFIP1*		Unknown
93		*NDN*		Unknown
94		*NIPA1*		Unknown
95		*MAGEL2*		Unknown
96		*LOC102724971*		Unknown
97		*SNURF*		Unknown
98		*NPAP1*		Unknown
99		*NIPA2*		Unknown
100		*TUBGCP5*		Unknown
101		*PWRN2*		Unknown
102		*SNORD116-1*		Unknown
103		*PWAR1*		Unknown
104		*IPW*		Unknown
105		*PWAR5*		Unknown
106		*SNORD115-1*		Unknown
107		*POTEB*		Unknown
108		*HERC2P2*		Unknown
109		*POTEB3*		Unknown
110		*NBEAP1*		Unknown
111		*PWARSN*		Unknown
112		*NF1P1*		Unknown
113		*SNORD116@*		Unknown
114		*GOLGA6L2*		Unknown
115		*IGHV1OR15-1*		Unknown
116		*POTEB2*		Unknown
117		*HERC2P6*		Unknown
118		*MIR4508*		Unknown
119		*NF1P2*		Unknown
120		*OR4N4*		Unknown
121		*PWAR6*		Unknown
122		*GOLGA6L22*		Unknown
123		*IGHV1OR15-3*		Unknown
124		*SNORD115-48*		Unknown
125		*SNORD115-44*		Unknown

126		*SNORD115-43*		Unknown
127		*SNORD115-42*		Unknown
128		*SNORD115-41*		Unknown
129		*SNORD115-40*		Unknown
130		*SNORD115-39*		Unknown
131		*SNORD115-38*		Unknown
132		*SNORD115-37*		Unknown
133		*SNORD115-36*		Unknown
134		*SNORD115-35*		Unknown
135		*SNORD115-34*		Unknown
136		*SNORD115-33*		Unknown
137		*SNORD115-32*		Unknown
138		*SNORD115-31*		Unknown
139		*SNORD115-30*		Unknown
140		*SNORD115-29*		Unknown
141		*SNORD115-26*		Unknown
142		*SNORD115-25*		Unknown
143		*SNORD115-23*		Unknown
144		*SNORD115-22*		Unknown
145		*SNORD115-21*		Unknown
146		*SNORD115-20*		Unknown
147		*SNORD115-19*		Unknown
148		*SNORD115-18*		Unknown
149		*SNORD115-17*		Unknown
150		*SNORD115-16*		Unknown
151		*SNORD115-15*		Unknown
152		*SNORD115-14*		Unknown
153		*SNORD115-13*		Unknown
154		*SNORD115-12*		Unknown
155		*SNORD115-11*		Unknown
156		*SNORD115-10*		Unknown
157		*SNORD115-9*		Unknown
158		*SNORD115-8*		Unknown
159		*SNORD115-7*		Unknown
160		*SNORD115-6*		Unknown
161		*SNORD115-5*		Unknown
162		*SNORD115-4*		Unknown
163		*SNORD115-3*		Unknown
164		*SNORD115-2*		Unknown
165		*MIR5701-1*		Unknown
166		*GOLGA6L6*		Unknown
167		*GOLGA6L1*		Unknown
168		*IGHV1OR15-5*		Unknown
169		*PWAR4*		Unknown
170		*SNORD116-18*		Unknown
171		*SNORD116-6*		Unknown

172		*SNORD116-21*		Unknown
173		*MIR1268A*		Unknown
174		*SNORD116-27*		Unknown
175		*SNORD116-26*		Unknown
176		*SNORD116-20*		Unknown
177		*SNORD116-14*		Unknown
178		*SNORD116-13*		Unknown
179		*SNORD116-7*		Unknown
180		*SNORD116-19*		Unknown
181		*GOLGA8DP*		Unknown
182		*ABCB10P1*		Unknown
183		*WHAMMP3*		Unknown
184		*GOLGA8CP*		Unknown
185		*SNORD116-29*		Unknown
186		*SNORD116-24*		Unknown
187		*SNORD116-22*		Unknown
188		*SNORD116-8*		Unknown
189		*SNORD116-5*		Unknown
190		*SNORD116-4*		Unknown
191		*IGHV4OR15-8*		Unknown
192		*SNORD116-28*		Unknown
193		*SNORD116-25*		Unknown
194		*SNORD116-23*		Unknown
195		*SNORD116-17*		Unknown
196		*SNORD116-16*		Unknown
197		*SNORD116-15*		Unknown
198		*SNORD116-12*		Unknown
199		*SNORD116-11*		Unknown
200		*SNORD116-10*		Unknown
201		*SNORD116-9*		Unknown
202		*SNORD116-3*		Unknown
203		*SNORD116-2*		Unknown
204		*GOLGA8IP*		Unknown
205		*SNORD109B*		Unknown
206		*OR4H6P*		Unknown
207		*GOLGA8S*		Unknown
208		*SNORD115-46*		Unknown
209		*SNORD116-30*		Unknown
210		*SNORD115-47*		Unknown
211		*SNORD115-45*		Unknown
212		*SNORD115-28*		Unknown
213		*SNORD115-27*		Unknown
214		*SNORD115-24*		Unknown
215		*LINC02203*		Unknown
216		*LINC01193*		Unknown
217		*HERC2P7*		Unknown

218		*SNORD64*		Unknown
219		*SNORD109A*		Unknown
220		*SNORD108*		Unknown
221		*SNORD107*		Unknown
222		*MIR5701-3*		Unknown
223		*MIR5701-2*		Unknown
224		*OR4M2*		Unknown
225		*IGHD1OR15-1A*		Unknown
226		*IGHD1OR15-1B*		Unknown
227		*IGHD2OR15-2A*		Unknown
228		*IGHD2OR15-2B*		Unknown
229		*IGHD2OR15-3A*		Unknown
230		*IGHD2OR15-3B*		Unknown
231		*IGHD2OR15-4A*		Unknown
232		*IGHD2OR15-4B*		Unknown
233		*IGHD2OR15-5A*		Unknown
234		*IGHD2OR15-5B*		Unknown
235		*BMS1P16*		Unknown
236		*MIR3118-2*		Unknown
237		*MIR3118-4*		Unknown
238		*MIR3118-3*		Unknown
239		*MIR4509-1*		Unknown
240		*IGHV1OR15-4*		Unknown
241		*IGHV1OR15-6*		Unknown
242		*OR4N3BP*		Unknown
243		*OR1Q1BP*		Unknown
244		*OR11K1BP*		Unknown
245		*OR4M2B*		Unknown
246		*OR4H6BP*		Unknown
247		*OR4N4C*		Unknown
248		*GOLGA8EP*		Unknown
249		*OR4N3P*		Unknown
250		*OR11K1P*		Unknown
251		*RPS27P2*		Unknown
252		*RPL5P1*		Unknown
253		*RPS8P10*		Unknown
254		*VN1R63P*		Unknown
255		*VN1R62P*		Unknown
256		*VN1R61P*		Unknown
257		*VN1R60P*		Unknown
258		*PWRN3*		Unknown
259		*LOC642131*		Unknown
260		*RN7SL545P*		Unknown
261		*RNU6-498P*		Unknown
262		*RN7SL536P*		Unknown
263		*RN7SL400P*		Unknown

264		*RNU6-1235P*		Unknown
265		*RNU6-749P*		Unknown
266		*RNU6-741P*		Unknown
267		*RNU6-631P*		Unknown
268		*RN7SL759P*		Unknown
269		*RN7SL495P*		Unknown
270		*RN7SL106P*		Unknown
271		*PWRN4*		Unknown
272		*LOC102724737*		Unknown
273		*LOC102723623*		Unknown
274		*LOC102723534*		Unknown
275		*NF1P9*		Unknown
276		*LOC100996379*		Unknown
277		*PDCD6IPP1*		Unknown
278		*MPHOSPH10P5*		Unknown
279		*MPHOSPH10P9*		Unknown
280		*MPHOSPH10P4*		Unknown
281		*GRAMD4P6*		Unknown
282		*GRAMD4P5*		Unknown
283		*BCAR1P2*		Unknown
284		*LONRF2P4*		Unknown
285		*LONRF2P3*		Unknown
286		*NBEAP4*		Unknown
287		*DMAC1P1*		Unknown
288		*OR11J6P*		Unknown

Supplementary Table 3: Point mutation identified on RCC metastases before treatment

	Patient 1	Patient 2	Patient 3	Patient 4
ABL1_pF359V_c1075T_G	**12**	**12**	**16**	**12**
NPM1_pW288fs12_c863_864insCATG_allele1	**13**	**9**	**9**	**11**
**NOTCH1_pL1575P_c4724T_C**	**11**	**21**	**0**	**9**
MEN1_p_c654_plus_3A_G	3	**20**	**10**	**11**
VHL_pE160K_c478G_A	**11**	1	**9**	**10**
RET_pM918T_c2753T_C	**28**	0	1	1
PTEN_pI101T_c302T_C	**17**	5	**14**	**11**
PTEN_pE235X_c703G_T	**14**	**12**	6	5
PTEN_pR173C_c517C_T	**19**	-1	7	3
PIK3CA_pC420R_c1258T_C	**16**	-1	-1	-1
PIK3CA_pC901F_c2702G_T	**15**	6	4	6
PIK3CA_pH701P_c2102A_C	**14**	-1	1	-1
PIK3CA_pY1021C_c3062A_G	**10**	5	8	8
PDGFRA_pD1071N_c3211G_A	**11**	1	**12**	8
NF1_pR816X_c2446C_T	**19**	**10**	**10**	5
NF1_pK1444E_c4330A_G	**19**	-1	7	5
NF1_pR1276X_c3826C_T	**16**	1	8	4
NF1_pR461X_c1381C_T	**12**	5	**12**	5
NF2_pQ362X_c1084C_T	**16**	1	**10**	4
RB1_p_c2107_minus_2A_G	**11**	-1	**13**	**9**
RB1_pQ702X_c2104C_T	**13**	6	**12**	**11**
RB1_pR787X_c2359C_T	**14**	**13**	8	-1
RB1_p_c1961_minus_1G_A	8	**9**	**12**	8
RB1_pR251X_c751C_T	7	6	**12**	0
CDKN2A_p_c151_min_1G_A	7	**11**	7	5
BRCA1_pG1077W_c3229G_T	**13**	-1	1	**14**
BRCA2_pR2678S_c8034G_T	**10**	**15**	**11**	-1
BRCA2_pS1682S_c5046T_C	**10**	3	**13**	**11**
APC_pQ1367X_c4099C_T	**15**	0	**14**	**10**
APC_pR213X_c637C_T	**15**	-1	0	7
APC_pQ789X_c2365C_T	**13**	-1	3	-1
APC_pE853X_c2557G_T	4	5	**12**	4
NPM1_pW288fs12_c863_864insTCTG_allele1	**19**	4	6	4
NPM1_pW288fs12_c863_864insCCTG_allele1	**11**	4	7	6
ERBB2_pG776S_c2326G_A	**12**	6	5	**10**
TP53_pE336X_c1006G_T	**10**	**11**	8	6
TP53_pC135F_c404G_T	-1	**11**	0	-1
KRAS_pQ61K_c181C_A	**9**	**14**	0	8
NRAS_pQ61H_c183A_C	**12**	6	3	-1
PAK7_pT397K_c1190C_A	**19**	**12**	4	6
SMAD4_pD537Y_c1609G_T	**16**	**11**	6	4
RUNX1_pR166X_c496C_T	**15**	8	4	8
MLH1_pC233R_c697T_C	**11**	-1	-1	4
IRAK1_pS690G_c2068A_G	**14**	-1	-1	**11**

TSHR_pM453T_c1358T_C	**13**	8	6	1
CBL_pR420Q_c1259G_A	**11**	7	**11**	5
MSH2_pR711X_c2131C_T	3	**11**	**9**	-1
INPP4A_pE940D_c2820A_C	**11**	-1	2	8

Supplementary Figure 2

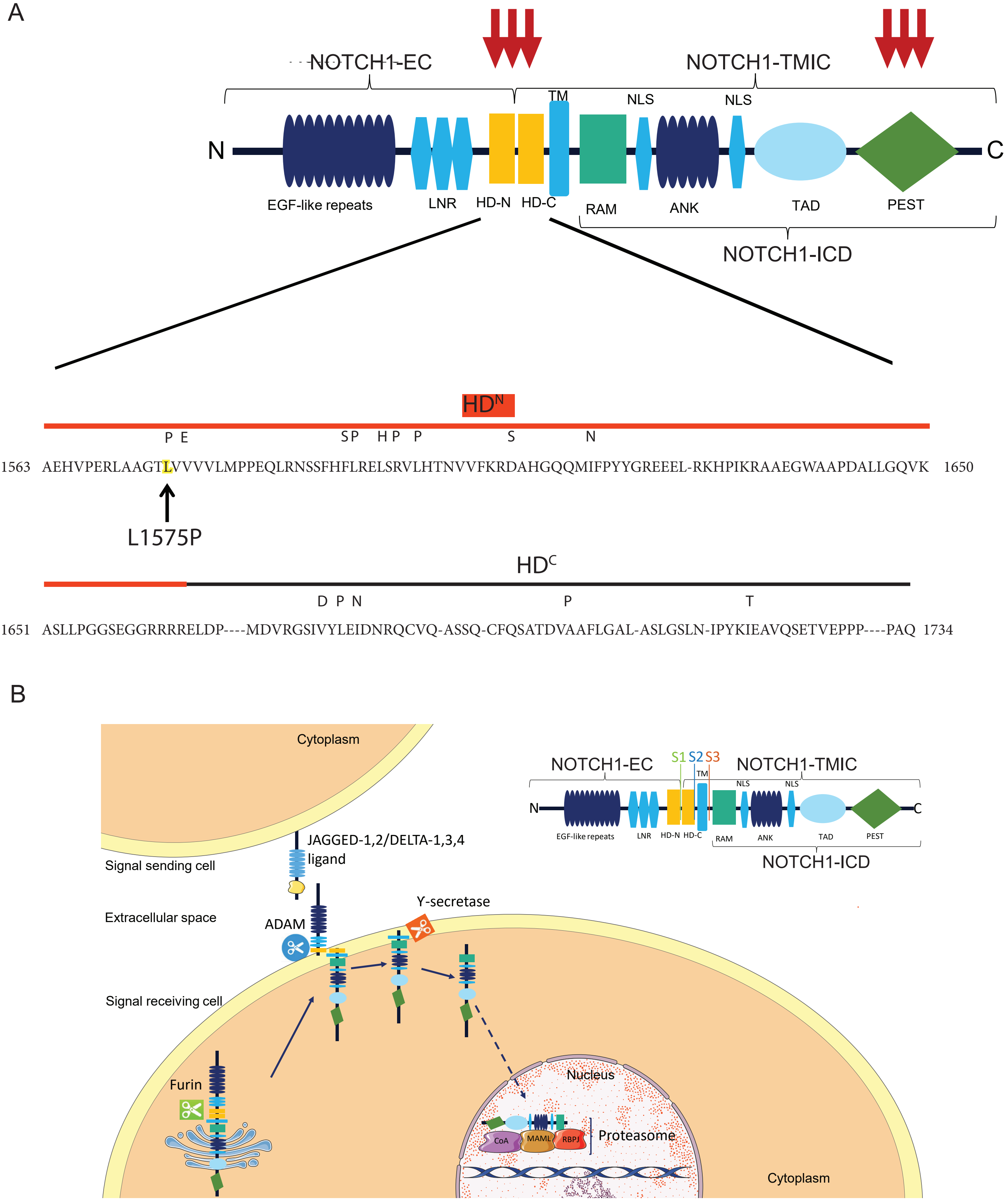

Supplementary Figure 3

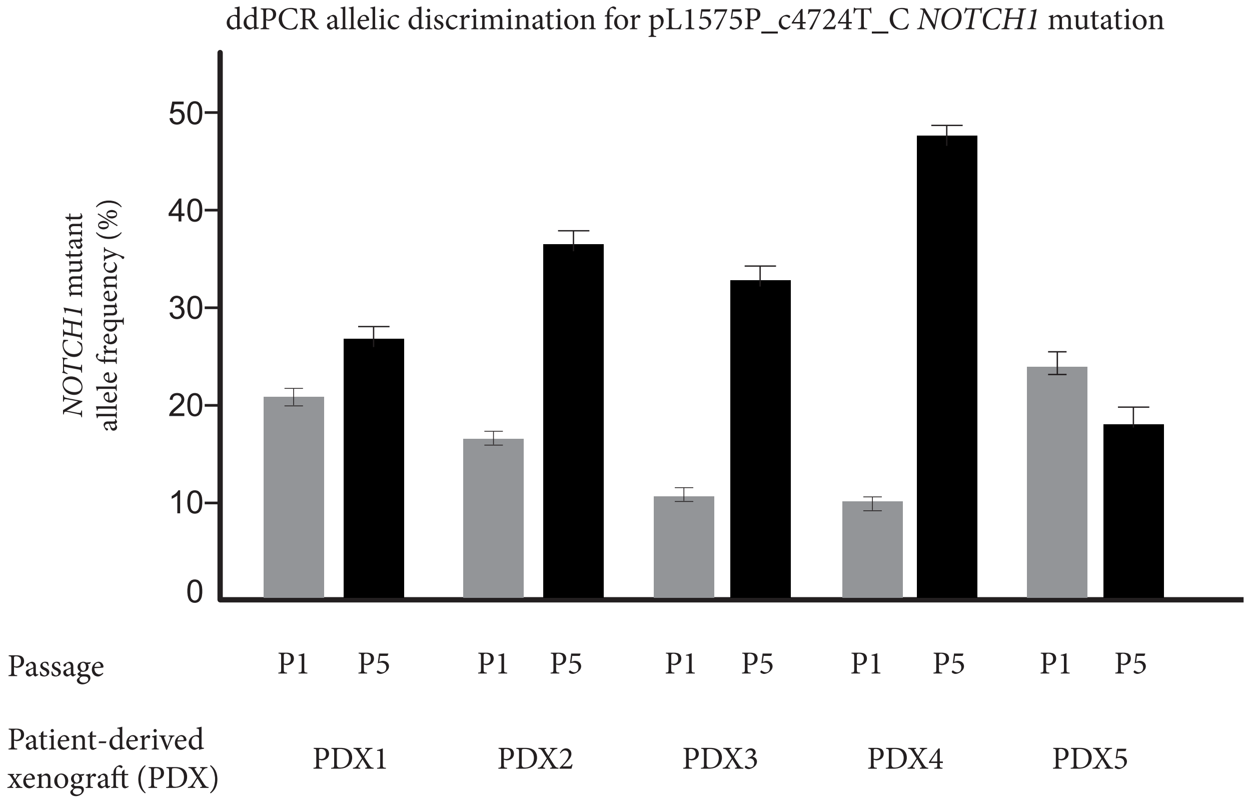

Supplementary Table 4. NOTCH1 targeted therapeutics

Mechanism of action	Drug name	Stage of drug development	IC50 (nM)
**Anti-DLL4 monoclonal antibodies**	Demcizumab	Phase II	
	Enoticumab (REGN421)	Phase I	
	MEdI0639	Phase I	
**Anti-NOTCH1 monoclonal antibodies**	Brontictuzumab	Phase I	
**γ-secretase inhibitors (GSI)**	RO4929097 (R4733)	Phase II	5
	LY3039478 (JSMD194)	Phase I	0.41
	Nirogacestat (PF-03084014)	Phase III	13.3
	AL101 (BMS-906024)	Phase II	1.6
	LY411575	Preclinical study	0.39
**NOTCH1 transcription complex inhibitors**	CB-103	Phase II	

Supplementary Table 5: Growth inhibition coefficient for drugs tested in XRCC model

XRCC Model	Drug	Growth inhibition coefficient
XRCC4	Untreated	0.92
	Sunitinib	-0.63
	CB103	-0.21
	Sunitinib+CB103	-0.65
XRCC5	Untreated	1.24
	Sunitinib	-0.60
	CB103	0.57
	Sunitinib+CB103	-0.62

Supplementary Figure 4

A

Patient (XRCC model 4)

Before treatment sunitinib

After treatment sunitinib

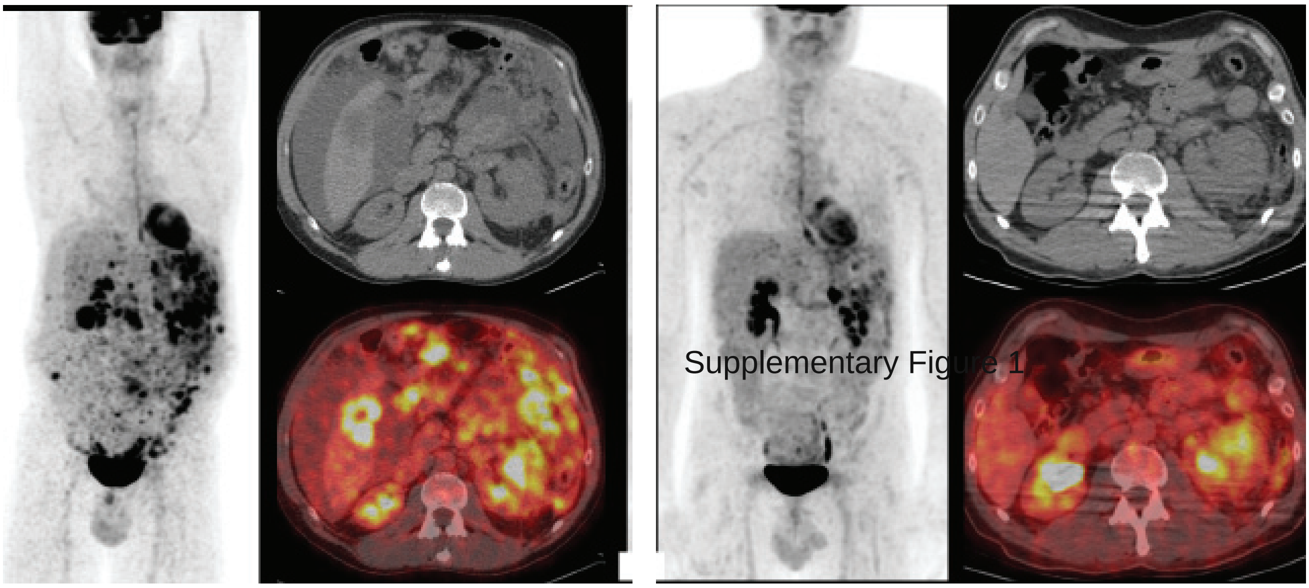

B

Patient (XRCC model 5)

Before treatment sunitinib

After treatment sunitinib

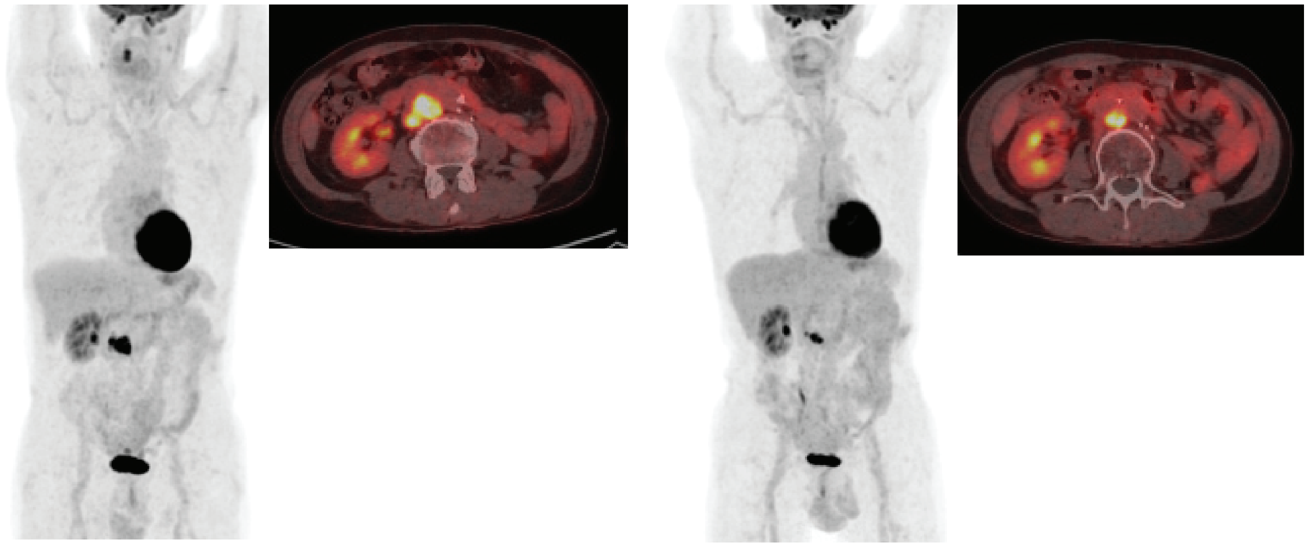

Supplementary Figure 5

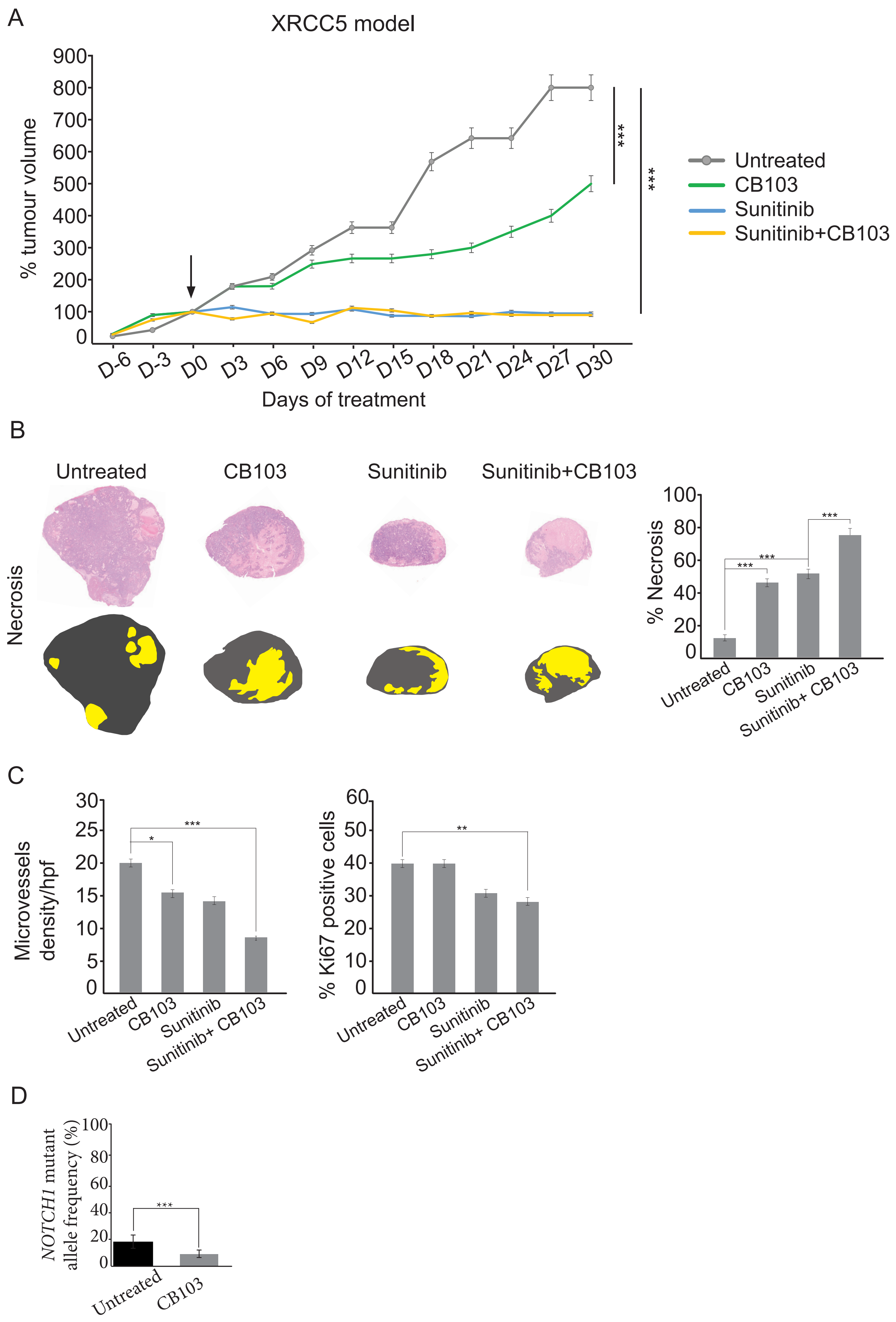

Supplementary Figure 6

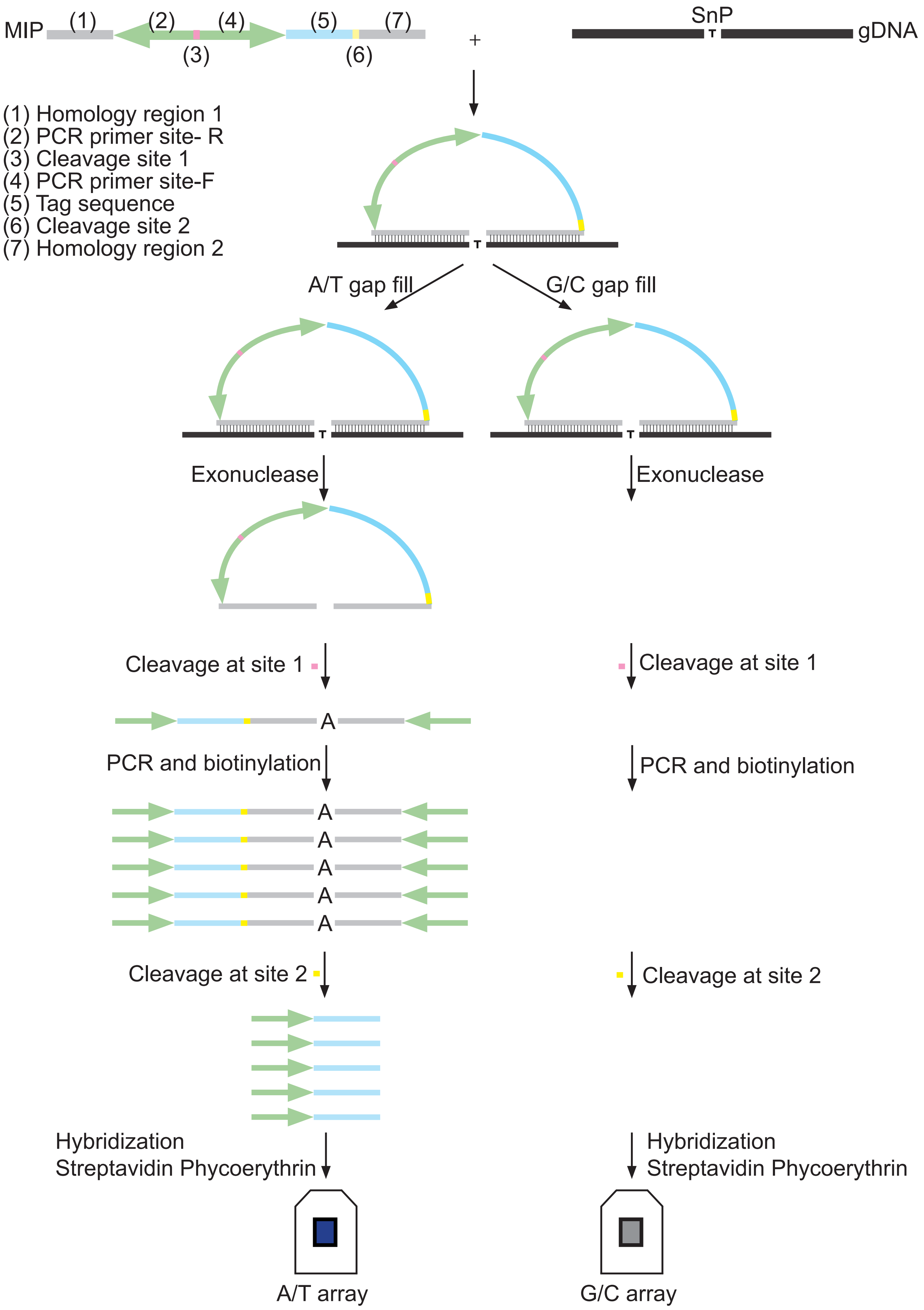

MIP: molecular inversion probe; SnP: Single nucleotid polymorphism; gDNA: genomic DNA

Supplementary Table 6: Characteristics of the five patient-derived xenograft models

	Sample from				
	Pre-treatment biopsy	Post-treatment biopsy	Histological type	Furhman grade	Metastatic localizations
HRCC1	+		Clear-cell	3	Lung, bone
HRCC2	+		Clear-cell	2	Lung
HRCC3		+	Clear-cell	4	Bone
HRCC4	+		Clear-cell	4	Lung, liver
HRCC5	+		Clear-cell	4	Lung

Supplementary Figure 7

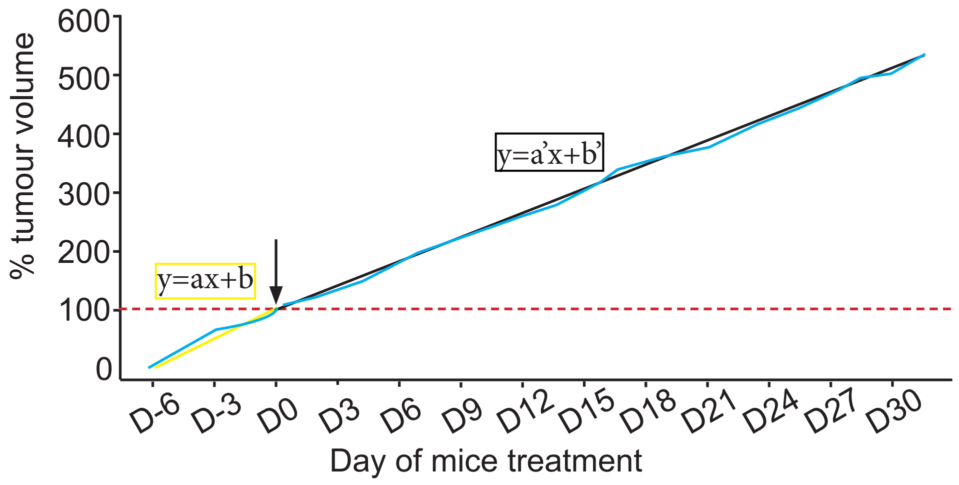

Supplementary File Excel 1

Assay External Id	Assay Name	0008231_A11	0008231_B02	0008231_B04	0008231_B06
MPL_pW515L_c1544G_T	amp725	4.715312	3.2156634	5.1703324	4.5344191
NRAS_pQ61H_c183A_C	tag100533	-1.0	11.831866	6.3235583	3.1857548
NRAS_pQ61P_c182A_C	tag100535	7.177846	5.2423573	9.2314558	4.1157255
NRAS_pQ61K_c181C_A	amp766	-1.0	-1.0	1.5148214	1.0298412
NRAS_pA18T_c52G_A	amp761	6.4878807	7.5783114	9.921361	2.0321229
NRAS_pG13D_c38G_A	tag100054	6.1034904	5.0451989	8.7258511	9.4866037
NRAS_pG12D_c35G_A	tag100116	2.8997612	3.6545827	0.46187055	4.725688
NRAS_pG12S_c34G_A	tag100117	0.39076245	2.8121858	8.7481365	9.5529995
CDC73_pW43X_c128G_A	amp574	3.1283891	5.7320142	3.2933941	1.3599352
ALK_pR1275Q_c3824G_A	tag110558	0.24737157	7.0684919	5.7840781	5.6996279
ALK_pF1245C_c3734T_G	tag110868	4.4777455	3.0023041	4.6037917	4.1525626
ALK_pF1245V_c3733T_G	tag110869	2.203809	1.6250321	2.8751299	2.2076585
ALK_pT1211T_c3633C_A	tag110564	3.366894	4.5347466	5.3966918	5.3614697
ALK_pF1174V_c3520T_G	tag110780	5.4898958	6.1730876	-1.0	8.5808907
ALK_pD1091N_c3271G_A	tag110829	3.4233489	4.8727627	10.493944	6.3311257
MSH2_p_c1276_plus_1G_A	tag111018	3.514323	7.460587	6.3377099	6.331315
MSH2_pR680X_c2038C_T	tag111013	-1.0	2.9952285	4.464025	0.48083764
MSH2_pR711X_c2131C_T	tag110706	-1.0	3.0463181	11.196543	9.2401018
MSH6_pP1082P_c3246G_T	tag100256	3.7965727	3.7674534	6.6736946	6.4557438
MSH6_pP1087fs3_c3261delC_allele1	tag100257	1.9423587	-1.0	-1.0	0.66997266
MSH6_pP1087fs5_c3261_3262insC_allele1	tag100528	-1.0	-1.0	1.2079544	-1.0
INPP4A_pE940D_c2820A_C	tag110899	7.6677766	10.904262	-1.0	1.6389618
VHL_p_c463_plus_2T_C	tag102376	2.9615827	1.854269	2.6671033	3.3214214
VHL_p_c464_minus_1G_A	amp922	-1.0	-1.0	-1.0	-1.0
VHL_pP61P_c183C_T	amp932	2.5798261	3.570842	4.7809787	1.9753727
VHL_pS65L_c194C_T	amp939	-1.0	2.8369431	-1.0	0.51232553
VHL_pS68X_c203C_A	amp940	-1.0	3.4724393	-1.0	1.4999263
VHL_pP81S_c241C_T	tag100125	6.2188892	6.1329684	7.5856524	5.2167525
VHL_pL85P_c254T_C	tag100080	4.8883162	2.8458502	4.0139437	3.5245829
VHL_pW88X_c263G_A	tag100085	2.6802289	1.7322558	2.5509775	1.3652266
VHL_pQ96X_c286C_T	tag100082	4.2267132	4.2103024	4.2072988	5.1037006
VHL_pG114C_c340G_T	tag100079	3.981343	1.5871989	3.7657022	3.7460265
VHL_pH115Y_c343C_T	tag100216	0.60130012	0.98010075	3.1019924	0.61070293
VHL_pW117R_c349T_C	tag110819	1.7763501	3.2312067	1.7085617	6.355628
VHL_pW117X_c350G_A	tag100219	4.6986504	-1.0	5.277534	4.613358
VHL_pL118P_c353T_C	amp928	-1.0	-1.0	3.4058876	-1.0
VHL_pQ132X_c394C_T	amp934	-1.0	-1.0	-1.0	3.786123
VHL_pI151S_c452T_G	tag100547	11.913697	7.7114406	7.454783	6.2152543
VHL_pL153P_c458T_C	amp929	3.408443	1.7382764	1.607164	2.085146
VHL_p_c463_plus_1G_T	tag102926	1.6795194	2.2557182	1.9121838	2.2615757
VHL_pE160K_c478G_A	tag100546	10.130236	10.970996	1.0460038	8.925602
VHL_pR161X_c481C_T	amp936	-1.0	-1.0	1.9941219	-1.0
VHL_pR167W_c499C_T	amp937	-1.0	-1.0	0.25089329	-1.0
VHL_pS183X_c548C_A	amp938	1.8521118	0.16115491	6.4049487	2.2966156
VHL_pL184P_c551T_C	amp930	2.1277673	2.4690557	1.4861472	5.1382179
VHL_pE189K_c565G_A	amp924	-1.0	0.12727816	0.62124145	1.8589697
TGFBR2_pR497X_c1489C_T	tag110763	4.9730673	6.1395645	8.2267647	8.6719913
MLH1_pC233R_c697T_C	tag110943	3.8034828	11.454423	-1.0	-1.0

MLH1_p_c790_plus_1G_A	tag110997	3.7624173	3.7535877	3.4283509	3.6038532
MLH1_pS556fs14_c1731G_A	tag111027	4.0125561	5.3834896	5.4289908	4.0502343
MLH1_p_c1732_minus_1G_A	tag110729	5.9260025	10.042222	3.953567	2.9745347
CTNNB1_pA5_A80del_c14_241del228_allele1	amp605	1.9301199	4.8439956	-1.0	-1.0
CTNNB1_pA13T_c37G_A	amp604	-1.0	-1.0	-1.0	0.67024857
CTNNB1_pD32N_c94G_A	tag100519	6.0162621	7.1839571	4.8743892	5.5870166
CTNNB1_pS33Y_c98C_A	amp609	4.0265732	-1.0	8.3706636	-1.0
CTNNB1_pG34E_c101G_A	tag100244	8.0938406	4.8835945	5.7653227	6.3918295
CTNNB1_pT41A_c121A_G	tag100144	5.9156423	4.3504357	4.7015004	6.1164355
CTNNB1_pT41I_c122C_T	tag100195	3.1362863	1.1824689	1.4571245	2.411267
CTNNB1_pS45P_c133T_C	tag100194	10.051488	7.6128893	1.30922	6.522615
PIK3CA_pR38H_c113G_A	tag102367	-1.0	5.5508652	-1.0	0.50824624
PIK3CA_pR88Q_c263G_A	amp792	6.672256	5.8552337	5.8208294	5.3703308
PIK3CA_pR108H_c323G_A	tag110892	8.0715122	5.0001864	5.9223542	5.4857678
PIK3CA_pG118D_c353G_A	amp784	-1.0	0.5508455	1.099442	2.3392167
PIK3CA_pC420R_c1258T_C	amp778	-1.0	15.961757	-1.0	-1.0
PIK3CA_pE453K_c1357G_A	amp780	2.8571703	8.7695971	1.4238058	7.4576912
PIK3CA_pE542K_c1624G_A	amp781	-1.0	-1.0	1.0634934	-1.0
PIK3CA_pE545K_c1633G_A	amp783	-1.0	3.669868	3.0529962	1.7880006
PIK3CA_pE545A_c1634A_C	tag101384	1.7083176	0.2657125	3.8822658	1.8572738
PIK3CA_pQ546K_c1636C_A	tag101886	2.6409543	4.2478385	5.1911955	8.0006332
PIK3CA_pH701P_c2102A_C	amp787	-1.0	14.093289	-1.0	0.93651354
PIK3CA_pC901F_c2702G_T	amp779	6.0806775	14.592525	5.7978086	3.7671022
PIK3CA_pY1021C_c3062A_G	tag101387	7.8100553	10.181917	5.3030701	7.8585086
PIK3CA_pT1025A_c3073A_G	amp793	0.88616407	4.4239106	0.036534164	5.2073321
PIK3CA_pT1025T_c3075C_T	amp794	5.8186097	3.1965568	3.6297941	6.1554461
PIK3CA_pM1043V_c3127A_G	tag102366	0.57031351	0.83434349	-1.0	0.68522811
PIK3CA_pM1043I_c3129G_T	tag103396	1.6290503	2.4951031	-1.0	3.7677848
PIK3CA_pH1047Y_c3139C_T	amp786	-1.0	-1.0	-1.0	-1.0
PIK3CA_pH1047R_c3140A_G	amp785	-1.0	1.1776801	-1.0	0.22875506
PIK3CA_pG1049S_c3145G_A	tag110926	2.7027984	2.8476224	4.861012	2.1303263
FGFR3_pR248C_c742C_T	tag100033	1.2982364	1.8008673	1.1191629	2.2031486
FGFR3_pS249C_c746C_G_allele1	tag100034	4.2813978	3.8383806	6.0761991	4.2250414
FGFR3_pA369A_c1107G_T	amp657	0.51996541	4.1657658	7.4216986	3.838763
FGFR3_pG370C_c1108G_T	amp659	-1.0	2.4750841	1.7360989	2.1779623
FGFR3_pS371C_c1111A_T_allele1	tag100103	2.6139414	0.72855145	1.8020378	5.1431317
FGFR3_pY373C_c1118A_G	tag100036	4.815362	3.0114238	4.0926218	0.7284286
FGFR3_pA391E_c1172C_A	amp658	3.8403976	5.6846552	2.5488622	3.0113261
FGFR3_pK650Q_c1948A_C	tag100201	4.0473475	2.922869	4.1281443	5.1193686
FGFR3_pK650M_c1949A_T_allele1	tag100253	0.099529997	0.19035867	1.1831228	0.80902618
FGFR3_pG697C_c2089G_T	amp660	-1.0	-1.0	-1.0	-1.0
PDGFRA_pN659K_c1977C_A	tag100537	7.0367689	9.5688009	4.2060709	8.0005379
PDGFRA_pT674I_c2021C_T	amp776	-1.0	-1.0	1.7472012	-1.0
PDGFRA_pF808L_c2422T_C	amp773	-1.0	0.5471285	-1.0	1.6745132
PDGFRA_pV824V_c2472C_T	tag100156	1.6946799	1.4743228	1.359647	2.9226305
PDGFRA_pD842Y_c2524G_T	tag100208	4.1080027	3.6669466	2.8921361	3.8935091
PDGFRA_pD842V_c2525A_T_allele1	tag100261	-1.0	-1.0	-1.0	-1.0
PDGFRA_pD846Y_c2536G_T	tag100536	2.7965903	3.6076233	4.4667506	9.6591377
PDGFRA_pN870S_c2609A_G	amp775	-1.0	-1.0	3.6297786	4.6304741

PDGFRA_pD1071N_c3211G_A	amp768	8.1166782	10.999471	0.56775302	11.560527
KIT_pV560D_c1679T_A_allele1	amp698	-1.0	3.4407701	-1.0	-1.0
KIT_pL576P_c1727T_C	amp693	2.4457047	-1.0	0.75908655	-1.0
KIT_pF584S_c1751T_C	amp690	1.1882986	2.7288616	-1.0	-1.0
KIT_pD52N_c154G_A	amp687	0.10192829	4.6117015	3.6956332	-1.0
KIT_pY503_F504insAY_c1509_1510insGCCTAT_allele1	amp705	-1.0	-1.0	-1.0	-1.0
KIT_pW557R_c1669T_C	tag102361	1.4186362	1.2925901	9.1129837	2.0112495
KIT_pW557_K558del_c1669_1674delTGGAAG_allele1	tag103393	2.4349031	2.6634824	3.8472943	3.4212525
KIT_pV559A_c1676T_C	tag102360	3.1365232	0.23514952	1.9334131	2.4389431
KIT_pP585P_c1755C_T	amp695	5.0711827	3.7318215	-1.0	3.6685567
KIT_pK642E_c1924A_G	tag100525	3.4372444	5.0802393	5.0186729	2.5857177
KIT_pV654A_c1961T_C	tag101882	3.3556929	1.5362495	4.6817985	3.6501477
KIT_pT670I_c2009C_T	amp696	-1.0	-1.0	-1.0	-1.0
KIT_pI798I_c2394C_T	amp691	0.19390063	2.0979249	1.8113177	2.0020506
KIT_pD816Y_c2446G_T	amp688	-1.0	0.21472915	0.90614569	0.24378289
KIT_pN822K_c2466T_G	tag103392	-1.0	-1.0	-1.0	-1.0
KIT_pY823D_c2467T_G	tag103395	3.5525565	1.5059268	0.77178103	0.10563457
KIT_pV825A_c2474T_C	amp701	2.4803984	2.5913918	2.6270554	1.8647524
KIT_pE839K_c2515G_A	amp689	-1.0	-1.0	2.0154369	2.6441789
NFKB1_p_c40_minus_1G_A	tag111038	4.9048414	4.3392534	3.8039694	1.6507534
FBXW7_pS582L_c1745C_T	amp654	-1.0	-1.0	3.0042706	-1.0
FBXW7_pR505C_c1513C_T	amp653	0.14483526	1.0869892	1.7518144	1.722743
FBXW7_pR479Q_c1436G_A	tag100250	5.7233233	8.6282282	3.6080718	7.2412219
FBXW7_pR465H_c1394G_A	tag101374	-1.0	4.311121	6.7530756	0.86324865
FBXW7_pR465C_c1393C_T	tag101878	3.7522552	5.1899581	2.4020441	0.67157382
FBXW7_pR393X_c1177C_T	amp649	0.50855368	2.2462275	3.138818	4.2195201
FBXW7_pR278X_c832C_T	tag101877	5.9552798	9.9434624	-1.0	4.2651691
FBXW7_pR224X_c670C_T	amp647	6.8153214	-1.0	-1.0	-1.0
PIK3R1_pG376R_c1126G_A	tag111045	2.396462	-1.0	-1.0	5.1980128
APC_pR213X_c637C_T	amp541	6.8793902	15.335615	-1.0	0.39951494
APC_pR232X_c694C_T	amp542	0.21506403	5.1993995	0.13696843	-1.0
APC_p_c835_minus_8A_G	tag103834	2.55022	1.9039376	0.76332653	-1.0
APC_pR283X_c847C_T	tag102907	3.9992604	6.3955097	5.4047165	5.9995017
APC_pR302X_c904C_T	amp544	-1.0	8.4363461	2.8463769	4.439518
APC_pR332X_c994C_T	amp545	3.0582268	2.9301331	3.3475432	3.1721537
APC_pR564X_c1690C_T	amp546	3.4968877	3.8044283	3.3832109	3.8126316
APC_pQ789X_c2365C_T	amp538	-1.0	12.948635	-1.0	3.0206339
APC_pE853X_c2557G_T	amp527	4.0834827	3.8900321	5.4938979	12.45909
APC_pR876X_c2626C_T	amp547	3.0505126	5.3630776	6.7435594	1.9887793
APC_pR1114X_c3340C_T	amp539	3.2608812	-1.0	1.7139647	-1.0
APC_pS1281X_c3842C_A	tag103835	2.7419441	4.4500012	4.8764057	3.87767
APC_pE1286X_c3856G_T	tag101869	4.6299319	5.4767933	1.3400291	6.031918
APC_pQ1291X_c3871C_T	tag101366	3.6345558	4.3280406	3.4629202	3.441828
APC_pQ1294X_c3880C_T	tag110857	1.6563731	2.2111874	3.9867039	2.0947223
APC_pE1309fs4_c3921_3925delAAAAG_allele1	tag102352	-1.0	-1.0	-1.0	-1.0
APC_pE1309fs6_c3923_3924insA_allele1	tag102905	0.21834746	0.3077679	-1.0	0.71069103
APC_pE1309X_c3925G_T	tag102906	2.649699	2.5815907	2.9156764	3.3845406
APC_pE1309fs4_c3927_3931delAAAGA_allele1	amp514	-1.0	-1.0	-1.0	-1.0
APC_pE1322X_c3964G_T	tag100185	1.3317435	4.1013451	3.1469066	5.8842015

APC_pQ1328X_c3982C_T	tag100516	3.0046873	4.6843629	5.5563679	6.195281
APC_pQ1338X_c4012C_T	amp531	-1.0	-1.0	-1.0	-1.0
APC_pS1341R_c4023T_G	tag100241	2.7032561	1.3583231	4.982688	3.1460266
APC_pE1345X_c4033G_T	tag100935	4.9574628	6.5366454	1.889539	1.8079422
APC_pE1353X_c4057G_T	tag100240	3.6553931	1.4643881	5.1967311	4.3034673
APC_pQ1367X_c4099C_T	tag100186	10.073657	15.155195	0.4921948	13.898117
APC_pQ1378X_c4132C_T	tag100517	3.7467687	2.270848	2.0424893	1.7787983
APC_pE1379X_c4135G_T	tag101363	1.3239744	1.701221	0.83877367	1.860945
APC_pQ1429X_c4285C_T	amp535	1.3412848	-1.0	7.0545053	-1.0
APC_pQ1447X_c4339C_T	tag100187	1.9748095	5.7886057	4.7981219	6.2905588
APC_pR1450X_c4348C_T	amp540	-1.0	-1.0	0.81115222	-1.0
APC_pE1451X_c4351G_T	tag100936	2.4327915	2.924382	4.0245795	6.243432
APC_pE1464X_c4390G_T	amp526	0.94225311	2.4215996	3.1903069	2.4079244
APC_pS1465fs3_c4393_4394delAG_allele1	tag100139	3.6186104	2.3371902	3.7384958	2.9039323
APC_pQ1469X_c4405C_T	tag100188	1.2930486	1.4207978	0.67695636	2.494729
APC_pG1499X_c4495G_T	amp528	-1.0	6.8949509	-1.0	5.0500059
APC_pT1537K_c4610C_A	amp552	-1.0	2.5699933	0.96146256	1.376701
APC_pT1556fs3_c4660_4661insA_allele1	tag101875	2.288094	1.162672	1.9105724	3.4987736
APC_pT1556fs3_c4666_4667insA_allele1	tag102908	3.2822046	3.4186351	0.6293813	3.1959176
APC_pE1577X_c4729G_T	tag111029	2.309252	2.9343965	1.8688974	4.4372606
APC_pE1397X_c4189G_T	amp523	4.1473598	4.6382451	5.4620128	4.0148191
APC_pQ1406X_c4216C_T	tag100136	1.5458071	0.36338162	2.1215911	3.2467895
APC_pE1408X_c4222G_T	amp524	-1.0	-1.0	-1.0	-1.0
CSF1R_pY969X_c2907T_G	tag100193	1.8068103	0.47227362	-1.0	0.54844928
CSF1R_pY969C_c2906A_G	tag100099	1.4146131	1.0592575	-1.0	2.3924537
CSF1R_pY969H_c2905T_C	amp602	1.4990162	2.2603347	0.78425193	3.0436773
CSF1R_pL301S_c902T_C	tag100142	5.968153	4.2591896	3.9746225	5.2421479
NPM1_pW288fs12_c863_864insCATG_allele1	tag101379	11.110229	13.289408	9.2349854	8.6655998
NPM1_pW288fs12_c863_864insCCTG_allele1	tag101380	6.0793452	10.774143	4.362699	6.5179377
NPM1_pW288fs12_c863_864insTCTG_allele1	tag101381	4.0119081	18.580753	3.5845828	6.4979801
EGFR_pR108K_c323G_A	amp637	1.6274734	-1.0	7.2979374	-1.0
EGFR_pT263P_c787A_C	amp640	0.82369918	-1.0	-1.0	-1.0
EGFR_pA289V_c866C_T	amp613	-1.0	-1.0	1.9162719	-1.0
EGFR_pL861Q_c2582T_A_allele1	tag100023	1.1502523	0.16874506	2.3226192	1.1569914
EGFR_pL861R_c2582T_G	tag110695	5.5922756	8.3274174	5.7331076	6.659976
EGFR_pG863D_c2588G_A	amp625	3.9516168	3.1254005	4.3633738	5.1880298
EGFR_pG598V_c1793G_T	amp623	0.011531563	5.6206093	5.1442919	6.592669
EGFR_pR677H_c2030G_A	amp638	3.7046366	1.331018	5.5539236	7.0208716
EGFR_pE709K_c2125G_A	tag102916	0.082146376	2.1767919	-1.0	0.54982334
EGFR_pE709A_c2126A_C	amp615	-1.0	3.0148728	0.83932555	-1.0
EGFR_pG719S_c2155G_A	tag100523	2.0171101	4.0565991	-1.0	3.1220157
EGFR_pG719C_c2155G_T	tag110607	0.48608652	2.3922338	3.0426564	4.465054
EGFR_pE746_A750del_c2235_2249del15_allele1	tag101372	2.8095853	1.6553614	2.5778377	2.3058782
EGFR_pE746_A750del_c2236_2250del15_allele1	amp619	3.7485301	1.5366725	-1.0	1.746617

EGFR_pE746_S752_V_c2237_2255_T_allele1	amp621	1.8472283	3.9947832	2.9710414	2.8347631
EGFR_pL747_A750_P_c2239_2248TTAAGAGAAG_C_allele1	amp626	-1.0	-1.0	1.2728218	0.26669416
EGFR_pL747_T751del_c2240_2254del15_allele1	amp630	-1.0	-1.0	-1.0	-1.0
EGFR_pL747_P753_S_c2240_2257del18_allele1	tag102359	3.1446867	3.4097483	4.6205111	7.6289039
EGFR_pD761Y_c2281G_T	tag100521	4.1719608	2.3625135	5.8142118	-1.0
EGFR_pS768I_c2303G_T	amp639	4.0304279	-1.0	-1.0	-1.0
EGFR_pV774M_c2320G_A	tag100029	-1.0	3.008472	2.7118821	0.23256268
EGFR_pT790M_c2369C_T	amp641	1.2825369	0.42156351	-1.0	6.164412
EGFR_pV819V_c2457G_A	amp643	-1.0	-1.0	1.6541238	-1.0
EGFR_pL833V_c2497T_G	amp632	1.2530204	4.3427191	-1.0	0.50444323
EGFR_pL858M_c2572C_A	tag100247	1.2014681	1.2544098	2.3694205	2.2754691
EGFR_pL858R_c2573T_G	tag100199	2.8902497	2.0311236	0.67410594	2.0439184
BRAF_pL618W_c1853T_G	tag110927	1.8213624	2.2215366	3.3710418	2.8774118
BRAF_pV600E_c1799T_A_allele1	tag100941	4.0156093	3.6189075	4.14921	5.4804411
BRAF_pV600K_c1798_1799GT_AA_allele1	tag101368	-1.0	-1.0	-1.0	-1.0
BRAF_pV600R_c1798_1799GT_AG_allele1	tag101370	0.93571705	0.090768978	-1.0	1.6360016
BRAF_pD594G_c1781A_G	tag101876	0.85514265	1.2250412	2.3922396	0.78724307
BRAF_pF583F_c1749T_C	tag110980	1.5963376	1.1983817	-1.0	-1.0
BRAF_pG469E_c1406G_A	tag102358	3.9620154	2.7542276	4.5330844	5.1129518
BRAF_pG469R_c1405G_A	tag102913	4.7827516	5.769496	7.1226788	6.536335
BRAF_pG466E_c1397G_A	tag102357	3.4098654	1.9104794	3.488512	2.4515209
BRAF_pG464E_c1391G_A	tag102356	5.2498136	4.7278366	5.4325113	3.055263
BRAF_pI326T_c977T_C	tag110747	2.3090792	1.8625298	2.6100481	2.1597972
FGFR1_pP252T_c754C_A	tag100030	2.3064866	2.590827	4.2366953	2.8671572
FGFR1_pS125L_c374C_T	tag100252	-1.0	3.6697099	5.2093663	4.7146297
IKBKB_pA360S_c1078G_T	tag110653	1.5254511	4.1863689	5.7105923	3.0519636
JAK2_pK191Q_c571A_C	tag110956	4.5902915	-1.0	-1.0	2.6174326
JAK2_pY570Y_c1710C_T	tag110659	-1.0	9.4524221	4.576097	2.1391671
JAK2_pV617F_c1849G_T	amp686	-1.0	2.8673549	2.5011857	-1.0
JAK2_pR683G_c2047A_G	tag110929	2.9006131	2.5519302	1.7111701	2.3969231
CDKN2A_p_c457_plus_2T_C	amp581	-1.0	2.8780866	-1.0	-1.0
CDKN2A_p_c457_plus_1G_T	tag100191	4.8647451	6.0700169	8.7165298	5.4539976
CDKN2A_pR131H_c392G_A	tag100015	1.4897457	1.7698549	2.2490716	1.8440312
CDKN2A_p_c150_plus_2T_C	tag100005	-1.0	7.1137905	-1.0	5.2120147
CDKN2A_pA30V_c89C_T	tag100006	2.9530604	3.251719	-1.0	4.3298812
CDKN2A_pE26X_c76G_T	tag100009	0.23163874	0.27537903	-1.0	0.02217019
CDKN2A_pW15X_c44G_A	tag100021	2.1446373	1.209826	2.6485987	2.2171378
CDKN2A_p_c1_minus_25C_T	tag100004	0.8927114	0.68273348	-1.0	0.35065103
CDKN2A_pE120K_c358G_A	tag100008	1.9416718	3.5064368	6.0823941	6.0885167
CDKN2A_pW110X_c330G_A	tag100020	1.2556474	1.3520632	2.337323	1.6933926
CDKN2A_pW110X_c329G_A	tag100019	3.0053649	4.5191555	8.8266935	4.6578565
CDKN2A_pD108N_c322G_A	tag100007	2.8684843	7.6408601	10.115907	7.3545609
CDKN2A_pE88E_c264G_A	tag100012	3.8223219	4.1103549	4.2181177	3.3410096
CDKN2A_pH83Y_c247C_T	amp590	1.7391677	1.8753943	-1.0	3.0815825
CDKN2A_pR80X_c238C_T	amp594	-1.0	1.2208723	-1.0	2.0021486
CDKN2A_pE69X_c205G_T	tag100011	2.0562224	2.1947	3.2518766	1.2053175
CDKN2A_pE61X_c181G_T	tag100010	1.1955116	-1.0	-1.0	2.5210226

CDKN2A_pR58X_c172C_T	tag100016	2.7450259	2.664448	4.8553066	2.5880895
CDKN2A_p_c151_minus_1G_A	tag100190	5.4098244	7.3096824	10.698084	6.5320048
CDKN2A_pP48L_c143C_T	tag100140	1.5357437	2.5414815	1.7135255	2.1156108
CDKN2A_pY44X_c132C_A	tag100141	1.4207458	-1.0	0.3250145	-1.0
CDKN2A_pS43I_c128G_T	tag100018	0.21299344	0.68616164	0.5296551	0.77851689
CDKN2A_pG35E_c104G_A	tag100013	1.7117289	0.58721602	1.0234272	2.04845
PTCH1_pW1018X_c3054G_A	amp799	-1.0	-1.0	-1.0	-1.0
PTCH1_pM561R_c1682T_G	tag100209	2.2891228	2.8896642	1.7064266	2.7073355
PTCH1_pQ417X_c1249C_T	amp798	-1.0	-1.0	-1.0	-1.0
PTCH1_pQ365X_c1093C_T	tag100265	1.1626531	4.1887078	6.222661	3.0967648
ABL1_pG250E_c749G_A	tag100002	0.3383365	-1.0	0.47679371	1.0894886
ABL1_pY253H_c757T_C	tag100003	2.3821316	1.5543668	3.8180478	4.3470154
ABL1_pE255K_c763G_A	amp501	2.6779854	2.6102099	4.4314089	3.785938
ABL1_pF311L_c931T_C	tag100932	1.0110316	0.98301983	1.155702	1.0779891
ABL1_pT315I_c944C_T	amp508	-1.0	-1.0	-1.0	-1.0
ABL1_pM351T_c1052T_C	tag100239	8.4482784	6.7246056	7.4589696	10.259717
ABL1_pE355G_c1064A_G	tag100931	3.3317695	2.976619	-1.0	4.7112117
ABL1_pF359V_c1075T_G	amp504	12.206912	12.168715	12.179368	15.640723
ABL1_pH396R_c1187A_G	tag100098	1.7882671	-1.0	2.0170276	1.1907475
NOTCH1_pQ2460X_c7378C_T	tag100115	6.1800966	9.594451	8.3859415	7.1513433
NOTCH1_pL1679P_c5036T_C	amp753	-1.0	2.2098563	-1.0	-1.0
NOTCH1_pL1601P_c4802T_C	tag100053	6.0505795	4.4104609	4.4741015	4.2219672
NOTCH1_pL1594P_c4781T_C	tag100114	3.8332214	1.2946441	3.8836215	3.5258496
NOTCH1_pL1586P_c4757T_C	tag100052	2.0388157	0.59755343	6.5952029	1.8427449
NOTCH1_pL1575P_c4724T_C	tag100051	8.8988752	11.46299	21.157406	0.30800349
RET_pC634R_c1900T_C	amp861	0.18909976	2.5509543	1.0369651	1.468172
RET_pC634Y_c1901G_A	tag100056	3.9867551	3.3244674	4.9566865	3.5750253
RET_pA664D_c1991C_A	amp860	7.8487358	-1.0	4.1788812	6.6748695
RET_pM918T_c2753T_C	amp863	1.0762515	28.379164	0.4172911	0.87443465
PTEN_pE7X_c19G_T	tag101388	3.83867	5.2607164	6.4623303	5.7798491
PTEN_pR15I_c44G_T	amp837	0.21982267	0.13176776	1.958859	3.7921784
PTEN_pQ17X_c49C_T	tag101895	5.2173271	7.6452417	1.1571163	7.6255131
PTEN_pL23F_c69A_C	tag101892	5.9895911	3.5821576	3.0607424	3.6960518
PTEN_pP38S_c112C_T	tag101893	2.5145977	3.127214	1.769383	2.6935389
PTEN_pL42R_c125T_G	tag100539	6.2779517	3.4731169	5.4840574	7.8632321
PTEN_p_c165_minus_2A_C	tag104692	9.3887053	10.152415	2.9285612	10.37822
PTEN_pH61R_c182A_G	tag104818	5.6496282	3.2202787	5.0587649	5.1216974
PTEN_pY68H_c202T_C	amp844	3.9527364	4.3153472	-1.0	3.2735472
PTEN_p_c209_plus_5G_A	tag104693	7.7644043	5.3938732	4.9132147	2.9302137
PTEN_p_c253_plus_1G_A	amp803	1.613973	3.6415157	-1.0	5.0836101
PTEN_p_c253_plus_1G_T	tag110963	6.2743516	2.7403085	-1.0	-1.0
PTEN_pH93Y_c277C_T	amp815	4.1796551	3.7879021	3.0092728	4.7270594
PTEN_pH93Q_c279T_G	amp814	4.1025286	-1.0	-1.0	-1.0
PTEN_pP95L_c284C_T	tag102371	2.2715237	2.4656527	3.0818932	4.4680634
PTEN_pI101T_c302T_C	tag101391	11.177982	16.775719	4.6410828	13.914928
PTEN_pD107Y_c319G_T	amp806	3.2321513	5.7856674	4.236351	3.2510374
PTEN_pQ110X_c328C_T	amp828	4.1983314	3.4152589	5.8522096	4.6311355
PTEN_pA126T_c376G_A	tag100266	4.5765944	5.2682443	0.70807135	4.4144769
PTEN_pG129R_c385G_A	amp810	7.0385723	1.7964718	-1.0	-1.0
PTEN_pR130X_c388C_T	amp836	0.97722471	3.2154267	0.84503305	2.5750287
PTEN_pR130Q_c389G_A	amp835	2.327548	4.7700853	-1.0	1.3839487
PTEN_pA151T_c451G_A	tag102923	7.3619432	5.2005992	2.5600033	4.8895268

PTEN_pY155C_c464A_G	tag100267	4.7425146	1.7659824	5.9213209	6.6655512
PTEN_pG165R_c493G_A	tag101890	8.2186365	6.0418129	3.7275736	7.6975784
PTEN_pQ171X_c511C_T	tag100540	6.0470204	9.0957823	0.20199156	7.4432244
PTEN_pR173C_c517C_T	amp838	3.284327	19.313326	-1.0	6.7416968
PTEN_pR173H_c518G_A	amp839	-1.0	-1.0	2.2258396	4.5127497
PTEN_pY174D_c520T_G	tag110875	4.9055228	1.5967996	-1.0	1.223527
PTEN_pQ214X_c640C_T	tag100957	4.9932947	3.3706989	6.6512527	0.16245227
PTEN_pQ219X_c655C_T	tag101393	5.7246895	6.8594012	5.8551421	4.8145204
PTEN_pR233X_c697C_T	tag100542	9.0803185	7.8464828	8.7181501	4.7567997
PTEN_pE235X_c703G_T	amp807	4.816524	13.704489	11.628378	5.5019631
PTEN_pQ245X_c733C_T	amp833	-1.0	-1.0	-1.0	0.97267723
PTEN_pP246L_c737C_T	tag101392	3.586293	0.42101896	4.5495157	5.5401487
PTEN_pG251C_c751G_T	tag101891	5.6176987	7.9137998	-1.0	6.7910557
PTEN_pK267fs9_c800delA_allele1	tag103839	7.9739084	6.1427927	7.6627884	7.3395553
PTEN_pW274X_c822G_A	tag104229	4.6508536	4.9625807	2.8655429	4.9167995
PTEN_pQ298X_c892C_T	amp834	-1.0	-1.0	9.5298119	-1.0
PTEN_pE299X_c895G_T	amp808	-1.0	-1.0	-1.0	-1.0
PTEN_pN323fs2_c968_969insA_allele1	tag104819	0.49020663	1.0641698	-1.0	1.7703191
PTEN_pN323fs21_c968delA_allele1	tag104821	1.0470986	1.0950314	1.446828	1.0125182
PTEN_pR335X_c1003C_T	tag103841	3.5070531	3.7739718	4.5068994	4.619235
PTEN_p_c1026_plus_1G_T	amp800	3.816103	5.8192167	4.8800759	5.2536316
PTEN_p_c1027_minus_2A_G	tag111023	6.8050981	7.8914495	9.0598259	4.6123962
FGFR2_pN549K_c1647T_G	tag110880	6.0147581	7.5673985	-1.0	8.6588736
FGFR2_pE475K_c1423G_A	tag110881	1.2342246	-1.0	-1.0	-1.0
FGFR2_pC382R_c1144T_C	tag110882	5.7857804	9.2213497	4.5091543	3.0363917
FGFR2_pY375C_c1124A_G	tag110800	0.83803833	4.5635982	-1.0	6.3565249
FGFR2_pK310R_c929A_G	tag110681	8.3483315	4.836112	7.5902162	6.3645191
FGFR2_pR203C_c607C_T	tag110728	-1.0	7.3333611	0.62955451	-1.0
HRAS_pQ61H_c183G_T	tag100041	1.1427735	1.2792161	2.5278351	2.1600988
HRAS_pQ61P_c182A_C	tag100043	2.3224895	3.0061436	2.3193045	6.3636413
HRAS_pQ61K_c181C_A	tag100042	0.99343824	1.7612363	2.2766242	3.1319346
HRAS_pG13D_c38G_A	tag100039	4.0865541	4.0209808	4.7709494	6.5383072
HRAS_pG13S_c37G_A	tag100040	0.64133716	3.7470844	5.045301	4.693758
HRAS_pG12D_c35G_A	amp679	6.0406032	4.3874216	4.847971	2.291028
HRAS_pG12S_c34G_A	tag100038	4.2826867	7.4213438	6.9243636	9.7063446
WT1_pR394W_c1180C_T	tag100127	4.4447794	2.5887558	0.62400228	2.6657155
WT1_pR390X_c1168C_T	amp943	-1.0	-1.0	-1.0	-1.0
WT1_pG379V_c1136G_T	tag110945	2.0511022	2.2323954	1.732111	2.1717517
WT1_pR362X_c1084C_T	tag110885	0.37593707	0.98181307	0.93152112	2.6025014
WT1_pS313X_c938C_A	tag110640	-1.0	0.26615152	0.82751161	0.64702117
WT1_pR301X_c901C_T	tag110776	3.6716416	2.2002177	0.71911854	4.2617269
WT1_pF154S_c461T_C	tag110755	-1.0	6.6108384	1.376102	1.9229527
WT1_pS46X_c137C_A	tag100086	1.1329031	-1.0	-1.0	-1.0
MEN1_pW471X_c1413G_A	tag100046	1.2535816	4.8108983	7.2509985	5.4657817
MEN1_p_c654_plus_3A_G	tag100044	10.811992	3.342823	20.180695	9.5618382
MEN1_pR98X_c292C_T	amp718	4.0528774	-1.0	-1.0	3.203716
ATM_pR337C_c1009C_T	tag110556	-1.0	2.5221877	-1.0	1.4035504
ATM_pR337S_c1009C_A	tag110559	-1.0	1.8820713	-1.0	-1.0
ATM_pV410A_c1229T_C	tag110939	3.1166046	3.3842905	1.1405059	0.33856678
ATM_pP604S_c1810C_T	amp558	2.2232351	0.81359965	1.1805286	3.5102947

ATM_pS707P_c2119T_C	tag111024	1.9401561	2.4169104	-1.0	0.74662018
ATM_pF858L_c2572T_C	amp557	2.2064955	2.5322068	3.1269369	1.9670857
ATM_pA1309T_c3925G_A	tag110600	1.1978571	0.39477766	5.6318154	2.6399846
ATM_pI1681V_c5041A_G	tag110877	2.7681978	7.0119982	-1.0	5.4044309
ATM_pQ2442P_c7325A_C	tag110796	1.183602	4.5587158	2.5455868	1.1411361
ATM_pR2443Q_c7328G_A	amp559	-1.0	0.95654786	3.1675062	5.1036477
ATM_pT2666A_c7996A_G	tag110994	4.1678772	3.4894631	2.9038458	1.4267812
ATM_pG2867E_c8600G_A	tag110942	-1.0	-1.0	5.8977313	5.8827281
ATM_pR3008C_c9022C_T	amp560	1.6099049	4.0586977	7.1071382	0.032016981
ATM_pR3008H_c9023G_A	amp561	-1.0	-1.0	-1.0	-1.0
ATM_pR3047X_c9139C_T	amp562	-1.0	4.6290483	3.0190291	-1.0
CBL_pY371H_c1111T_C	tag110995	0.46986869	5.1789341	-1.0	4.3840289
CBL_pV391I_c1171G_A	tag110878	0.76087421	3.8086128	2.5267026	4.8693733
CBL_p_c1228_minus_2A_G	tag110799	3.0143268	1.8443608	5.319056	3.8808548
CBL_pR420Q_c1259G_A	tag110879	5.0868039	11.394435	7.0315576	10.561402
KRAS_pA146T_c436G_A	amp708	-1.0	-1.0	1.7506852	-1.0
KRAS_pQ61H_c183A_C	tag100204	4.1912932	6.8515077	1.7886328	8.4905157
KRAS_pQ61P_c182A_C	tag100154	5.8598995	6.0760899	1.6073655	7.5892248
KRAS_pQ61K_c181C_A	amp715	8.4653759	9.39291	14.435107	0.2021064
KRAS_pA59T_c175G_A	amp709	2.5708971	-1.0	-1.0	0.46460411
KRAS_pG13D_c38G_A	amp712	3.1482871	3.9378211	2.251389	5.2750626
KRAS_pG13S_c37G_A	tag100106	2.0763445	0.053069931	0.25576732	-1.0
KRAS_pG12D_c35G_A	tag100152	4.5467324	5.0279102	4.8103886	4.1520524
KRAS_pG12S_c34G_A	tag100153	-1.0	-1.0	7.743804	-1.0
PTPN11_pG60V_c179G_T	amp849	4.0000615	2.3982854	2.4293981	2.3624213
PTPN11_pD61Y_c181G_T	amp846	2.7428596	1.9980229	3.7671511	3.7313683
PTPN11_pA72T_c214G_A	amp845	-1.0	-1.0	-1.0	-1.0
PTPN11_pE76K_c226G_A	tag101396	2.9892204	5.8623343	3.5918603	7.1727109
PTPN11_pS502P_c1504T_C	tag100118	6.2668552	8.3912859	6.5238733	7.5313096
PTPN11_pG503V_c1508G_T	tag100158	3.1183329	-1.0	3.6353574	1.636174
HNF1A_pW206L_c617G_T	amp678	-1.0	1.0964133	-1.0	-1.0
HNF1A_pW206C_c618G_T	tag100150	4.2171078	4.6315088	3.9305677	5.786479
FLT3_pD835E_c2505T_G	tag100948	1.3811764	1.3090899	4.1583219	3.5111737
FLT3_pD835V_c2504A_T_allele1	tag101375	7.8998733	6.4126415	3.8403051	0.65960544
FLT3_pD835N_c2503G_A	tag101879	3.8428545	2.7995455	4.1129847	3.0501354
FLT3_pL561L_c1683A_G	amp674	-1.0	-1.0	2.0248218	0.89983338
BRCA2_pR18H_c53G_A	tag111019	1.2547346	-1.0	0.45327356	-1.0
BRCA2_pE187K_c559G_A	tag110830	0.16803387	-1.0	-1.0	0.15122327
BRCA2_pT630I_c1889C_T	tag110870	1.6111081	5.0487971	3.1481924	-1.0
BRCA2_pP920S_c2758C_T	tag110933	-1.0	5.1689649	8.3979425	6.154789
BRCA2_pI1017S_c3050T_G	tag110901	4.1522031	9.2744923	-1.0	8.3246937
BRCA2_pG1338G_c4014C_T	tag111033	4.8159943	3.9114017	3.7748053	7.2073131
BRCA2_pE1593X_c4777G_T	tag110902	3.3670585	2.723928	3.008358	5.3947144
BRCA2_pS1682S_c5046T_C	tag110598	10.805107	10.242161	2.7483714	12.683793
BRCA2_pV1988I_c5962G_A	tag110609	-1.0	2.7194493	3.860399	0.65190262
BRCA2_pH2415N_c7243C_A	tag110626	2.5428014	1.6497654	2.9774606	3.3341763
BRCA2_pR2678S_c8034G_T	tag110959	-1.0	10.159115	14.651917	11.156391
BRCA2_pR2787H_c8360G_A	tag110592	2.6879289	1.2392507	2.5280139	0.018753842
BRCA2_pR2842C_c8524C_T	tag111014	3.6016223	9.9375	3.1841011	4.9018173
BRCA2_pQ2934X_c8800C_T	tag110985	6.933641	0.57287472	-1.0	4.9078822
BRCA2_pD3095E_c9285C_A	tag110606	1.4314215	6.652616	6.0841994	6.2951789
BRCA2_pI3103M_c9309A_G	tag110645	5.6876416	-1.0	2.1910319	1.303641

BRCA2_pR3128X_c9382C_T	tag110593	2.1478226	6.2456193	7.5950751	7.4034481
RB1_pE54X_c160G_T	tag110986	-1.0	5.2309518	6.417531	2.6634381
RB1_pS82X_c245C_A	tag110934	2.1181235	8.5939217	4.1031675	0.86738658
RB1_p_c380_plus_1G_A	tag110743	4.1406803	3.4056935	6.1307111	3.996191
RB1_pE137X_c409G_T	amp853	2.9886854	2.3103228	4.6616693	-1.0
RB1_pQ217X_c649C_T	tag110960	2.8591058	2.7502103	0.16446117	4.3687701
RB1_pR251X_c751C_T	tag110831	0.3293134	6.6232886	5.629643	12.371955
RB1_pR320X_c958C_T	amp854	-1.0	-1.0	-1.0	-1.0
RB1_pR358X_c1072C_T	amp855	-1.0	6.1359415	3.5826154	5.2610106
RB1_pQ395X_c1183C_T	tag110637	-1.0	3.5078981	8.0180254	-1.0
RB1_p_c1215_plus_1G_A	amp851	-1.0	-1.0	-1.0	-1.0
RB1_pE440X_c1318G_T	tag110620	1.1571538	3.9821332	-1.0	9.4375782
RB1_pR445X_c1333C_T	tag111020	4.4901438	3.6112013	2.5587659	6.3619204
RB1_pR455X_c1363C_T	amp856	1.5634054	6.2511315	5.8093634	3.9290056
RB1_p_c1499_minus_1G_T	tag104842	-1.0	-1.0	-1.0	2.2931967
RB1_pR552X_c1654C_T	tag104230	0.92421442	2.0150666	2.5146451	2.117538
RB1_pR556X_c1666C_T	tag101897	-1.0	0.2828919	3.5485628	2.3073046
RB1_pR579X_c1735C_T	amp859	0.68801588	6.7851896	6.7133331	9.6190376
RB1_p_c1961_minus_1G_A	tag110904	7.867053	7.9489975	8.5878305	12.012525
RB1_pQ685X_c2053C_T	tag110784	-1.0	4.013752	5.4545093	5.4177403
RB1_pQ702X_c2104C_T	tag110649	11.201917	13.032303	5.5824814	12.015815
RB1_p_c2107_minus_2A_G	tag111042	9.1658831	10.596729	-1.0	13.489717
RB1_pE748X_c2242G_T	tag110625	4.8609915	4.0306535	3.774087	3.2172129
RB1_p_c2326_minus_2A_C	tag110871	7.4302096	9.0563622	-1.0	10.662297
RB1_pR787X_c2359C_T	tag110872	-1.0	14.410912	12.722295	7.8245034
TSHR_pM453T_c1358T_C	amp918	0.57215446	12.56707	8.2162628	6.2711711
TSHR_pA623S_c1867G_T	tag100960	-1.0	4.8365779	2.8674498	3.0705907
TSHR_pA623V_c1868C_T	tag101398	1.1538335	2.9937618	2.3411024	2.6565018
TSHR_pI630L_c1888A_C	tag100545	3.3963213	3.9102638	1.0826309	3.6389647
TSHR_pT632I_c1895C_T	tag100273	-1.0	1.0730473	-1.0	1.1820229
CDH1_p_c1009_minus_1G_A	amp575	-1.0	8.4864397	-1.0	3.1575222
CDH1_pI374I_c1122C_T	amp576	-1.0	-1.0	-1.0	-1.0
TP53_pR342X_c1024C_T	tag100124	4.6690612	4.5891628	7.055418	5.8290668
TP53_pE336X_c1006G_T	tag100067	5.6720743	10.180836	11.376378	7.7390614
TP53_pQ331X_c991C_T	tag110908	-1.0	4.6277184	3.7990813	6.2249703
TP53_p_c920_minus_1G_A	tag110798	0.52675718	6.7346411	0.14673744	3.4805758
TP53_pR306X_c916C_T	tag100123	1.4044298	0.90023166	0.04221807	3.1412628
TP53_pE298X_c892G_T	tag100066	4.248929	3.1944499	4.56564	4.4834361
TP53_pE285K_c853G_A	tag100122	2.9213793	5.4176669	8.3620996	5.0256343
TP53_pR273H_c818G_A	tag100169	-1.0	3.6189995	7.2830973	7.6075969
TP53_pR273C_c817C_T	tag100168	0.43075839	1.7194084	-1.0	0.39012143
TP53_pG266E_c797G_A	tag100544	4.1062474	5.6293912	5.8798137	5.3034511
TP53_p_c782_plus_1G_T	tag110665	1.0355332	4.4994521	4.1817045	3.3880868
TP53_pR249S_c747G_T	amp906	6.1178908	3.5928776	1.5832576	2.8855774
TP53_pR248Q_c743G_A	amp904	2.2423937	3.3688345	2.0112553	1.3139342
TP53_pR248W_c742C_T	amp905	3.2289357	1.9620948	1.3387623	1.3975544
TP53_pG245S_c733G_A	amp894	-1.0	1.6580788	-1.0	0.27384466
TP53_pY236C_c707A_G	tag100959	4.9815731	3.8693662	0.40086558	5.2422695
TP53_p_c672_plus_1G_A	tag100120	3.461297	5.6297154	3.7547016	5.02283
TP53_pY220C_c659A_G	amp913	5.0176845	2.4824092	3.927141	5.9053087
TP53_pR213X_c637C_T	tag101899	0.5808726	2.7105217	2.7651699	0.20738657
TP53_pR196X_c586C_T	tag100272	2.4448245	4.1696272	6.5018291	5.4076343
TP53_pH193R_c578A_G	tag100165	7.4262443	7.9942975	5.1129861	5.8320007

TP53_pH179Q_c537T_G	tag100069	3.1567585	2.7140312	3.0641873	2.5535314
TP53_pH179R_c536A_G	tag100070	3.3642783	2.641923	3.7286625	3.6136689
TP53_pC176F_c527G_T	amp890	2.7410109	3.4028606	3.9720366	1.741888
TP53_pR175H_c524G_A	amp901	4.0594683	4.3765812	2.4448669	5.2955904
TP53_pY163C_c488A_G	tag100076	3.6559293	1.3636528	4.8111734	5.0878849
TP53_pA159V_c476C_T	amp888	2.8108935	-1.0	-1.0	1.1494712
TP53_pR158H_c473G_A	amp900	-1.0	-1.0	-1.0	-1.0
TP53_pV157F_c469G_T	tag100075	2.0949857	1.5506772	3.0953913	0.48994318
TP53_pC135F_c404G_T	amp889	-1.0	-1.0	10.8249	0.2931025
TP53_pK132Q_c394A_C	tag100166	7.9460382	1.7534473	2.759881	7.9955525
TP53_p_c376_minus_1G_A	tag100164	0.91955572	0.21919431	6.4496436	0.30897111
TP53_pC124R_c370T_C	tag110632	-1.0	6.043571	-1.0	2.9957459
TP53_pF113C_c338T_G	tag110809	1.7987372	6.699975	4.6381278	0.5478251
MAP2K4_pR154W_c460C_T	tag110983	1.8923535	5.7195621	-1.0	3.1675777
MAP2K4_pS184L_c551C_T	tag111040	1.088306	2.2286518	-1.0	-1.0
MAP2K4_pS280X_c839C_A	tag110782	6.2180924	4.4494405	0.73040265	1.3716114
NF1_pR304X_c910C_T	tag110883	6.2241426	4.324451	4.7192526	2.6835914
NF1_pR461X_c1381C_T	tag110730	4.6196046	11.901588	5.3255477	12.023381
NF1_pR816X_c2446C_T	tag110724	4.7334743	19.152634	9.8961411	9.8484945
NF1_pR1276X_c3826C_T	tag110686	4.3620949	16.298796	1.0498688	8.4294605
NF1_pR1362X_c4084C_T	tag110848	2.523139	1.1436357	6.0622554	0.75196725
NF1_pK1444E_c4330A_G	tag110708	5.2260284	19.305214	-1.0	7.3749599
NF1_pR1769X_c5305C_T	tag110802	2.0901594	2.4656746	1.661424	1.8950512
NF1_pR1968X_c5902C_T	tag110944	3.9952362	3.9690356	0.62144852	0.22627632
ERBB2_pL755S_c2264T_C	tag100249	4.7418022	4.1815057	6.1185303	5.156971
ERBB2_pG776S_c2326G_A	amp644	9.8563499	12.453215	6.2932916	5.0125136
ERBB2_pV777L_c2329G_T	amp646	5.7334862	3.7481174	-1.0	0.92754215
BRCA1_p_c5278_minus_1G_T	tag110749	2.3284802	3.4192643	2.9823449	4.0983052
BRCA1_pR1751X_c5251C_T	tag110751	4.0067396	4.8707967	7.2206306	-1.0
BRCA1_pE1725X_c5173G_T	tag110931	3.59233	4.2243147	2.7636936	-1.0
BRCA1_pS1140G_c3418A_G	tag110783	2.7284782	1.642206	1.4368649	1.9537317
BRCA1_pG1077W_c3229G_T	tag110900	13.528924	13.283318	-1.0	0.85309803
BRCA1_pS1009X_c3026C_A	tag110588	2.1375146	1.859238	2.1734502	3.2509398
BRCA1_pG778C_c2332G_T	tag110932	2.7396312	6.2776871	1.8023182	7.3353281
BRCA1_pH448H_c1344C_T	tag111031	3.8127255	2.0896764	6.279201	6.4503789
BRCA1_pW372X_c1116G_A	tag110958	1.1250594	4.0261655	0.89551419	7.141212
BRCA1_p_c134_plus_1G_T	tag110984	4.8654151	5.6473522	5.4430938	6.6511965
BRCA1_pL30F_c90G_T	tag110753	-1.0	3.6466784	4.8444657	0.10390439
SMAD4_pQ245X_c733C_T	amp870	1.1041347	1.2209883	-1.0	2.4110897
SMAD4_pE330A_c989A_C	tag110884	1.3881317	2.4589133	6.6184711	3.3447163
SMAD4_pY353C_c1058A_G	tag110850	1.9059845	1.7593825	1.3798389	1.1205254
SMAD4_pG358X_c1072G_T	tag110804	0.080659717	5.3876657	3.7683775	3.3504064
SMAD4_pR361H_c1082G_A	tag110852	3.5984054	3.0999043	3.4342017	3.1201849
SMAD4_pR445X_c1333C_T	amp871	4.4232526	1.8999903	3.727443	-1.0
SMAD4_pR497H_c1490G_A	tag110853	2.4550812	1.3724117	0.30851042	1.4432389
SMAD4_pG168X_c502G_T	tag110849	-1.0	3.2225525	3.2200499	6.7039127
SMAD4_pK507Q_c1519A_C	tag110805	6.6855659	6.5328431	1.0676578	0.47039035
SMAD4_pD537Y_c1609G_T	tag110762	4.1211457	16.210037	10.707551	6.487329
STK11_pQ37X_c109C_T	amp885	0.37943235	-1.0	8.4306345	0.10799982
STK11_pQ170X_c508C_T	amp884	0.69212902	-1.0	6.8543348	-1.0
STK11_pD194Y_c580G_T	amp882	-1.0	1.7370569	7.9935069	1.4104029
STK11_pP281L_c842C_T	tag100064	0.69333029	1.8636842	0.13290907	1.5123866
PAK7_pT397K_c1190C_A	tag110707	6.3739386	18.516918	11.537224	3.6564898

SRC_pQ531X_c1591C_T	amp881	0.35372543	-1.0	8.2788544	-1.0
RUNX1_pR204Q_c611G_A	tag100213	6.4197454	5.0778594	2.1118488	4.1316586
RUNX1_pD198G_c593A_G	tag100211	0.089172021	2.0434077	1.8225203	1.9695089
RUNX1_pD198N_c592G_A	tag100212	2.9245429	5.3798356	5.1181078	4.4657698
RUNX1_pR166X_c496C_T	amp868	7.9719615	15.118157	8.1100492	4.3597007
RUNX1_pI114I_c342C_A	tag100159	1.7635694	1.4945331	3.8001251	2.1603661
RUNX1_pR107C_c319C_T	tag100057	2.5901344	5.8913918	-1.0	6.1711473
SMARCB1_pR40X_c118C_T	tag101397	0.40120849	-1.0	0.6196627	-1.0
SMARCB1_pY47X_c141C_A	tag100271	3.0957036	9.6250029	8.8381319	7.9949389
SMARCB1_pR158X_c472C_T	amp874	3.8784666	3.9466274	2.5790396	2.0485017
SMARCB1_pR201X_c601C_T	amp875	2.7376583	9.3502445	3.534363	-1.0
SMARCB1_pE216X_c646G_T	amp873	5.7125163	-1.0	4.9056149	4.903441
SMARCB1_pS299S_c897G_A	amp878	0.21383396	-1.0	4.5124397	3.8173571
SMARCB1_p_c1119_minus_41G_A	amp872	2.0166063	1.3157719	3.7062922	2.9616647
SMARCB1_pR377H_c1130G_A	amp876	-1.0	-1.0	1.7946529	-1.0
NF2_pR57X_c169C_T	tag100207	3.2184899	1.9729131	3.4280086	3.2246654
NF2_p_c240_plus_2T_C	amp732	-1.0	-1.0	-1.0	-1.0
NF2_p_c516_plus_1G_A	amp733	-1.0	3.4993668	4.8284397	2.6528695
NF2_pR196X_c586C_T	tag100049	6.3220868	8.4295807	6.8795328	9.126462
NF2_pR198X_c592C_T	tag100050	1.7611485	6.5890408	4.5821958	6.7348537
NF2_pQ212X_c634C_T	tag100951	4.6801887	3.7732131	-1.0	0.41143984
NF2_pV219M_c655G_A	amp748	2.1276748	3.3374593	-1.0	4.3777266
NF2_p_c675_plus_1G_T	tag100530	2.0182121	6.2355156	-1.0	8.0672474
NF2_pR262X_c784C_T	tag101378	4.6961761	3.5791886	-1.0	-1.0
NF2_p_c810_plus_2T_C	tag100206	2.9891243	1.9151031	4.2593565	4.00314
NF2_pQ337X_c1009C_T	amp737	0.55827647	-1.0	1.6816338	0.86957073
NF2_pR341X_c1021C_T	tag100112	0.68054056	1.3968507	-1.0	2.2511213
NF2_pQ362X_c1084C_T	amp738	4.3059773	16.361784	1.3317742	10.327072
NF2_pQ400X_c1198C_T	tag100110	2.2838862	1.9341257	3.291693	4.5615616
NF2_pQ410X_c1228C_T	tag100111	2.7258012	3.2781615	2.4012265	3.4061589
NF2_p_c1340_plus_1G_A	amp731	1.8504249	4.1237454	4.0757775	1.8108997
NF2_pQ456X_c1366C_T	tag100048	6.5683146	9.7045498	6.6770101	10.950913
NF2_pR466X_c1396C_T	tag100113	-1.0	-1.0	-1.0	-1.0
GATA1_pQ17X_c49C_T	amp675	3.6640022	1.7551972	2.889276	3.1586518
GATA1_pV74I_c220G_A	tag100255	3.4568496	6.3149214	6.2690678	6.4402103
AR_pA749A_c2247C_T	tag110633	0.81764984	1.4689652	3.2613044	1.6614983
IRAK1_pS690G_c2068A_G	tag110610	11.274024	13.589069	-1.0	-1.0

Supplementary Word 1

***NOTCH1* mutation analysis**

NOTCH1 mutation analysis was performed by direct sequencing of reverse transcription (RT)–PCR–amplified *NOTCH1* transcripts. Tumor samples were analyzed by direct sequencing of PCR products expanding *NOTCH1* exons encompassing the sequences encoding the HD domains.

Primer sequences were as follows:

exon FW: GACCAGTACTGCAAGCACCA

exon RV: CAGGAAGTGGAAGGAGCTGT

Sonde *NOTCH1* 12772 c.4724T>C p.L1575P
